# Diversification of the aquaporin family in geographical isolated oyster species promote the adaptability to dynamic environments

**DOI:** 10.1186/s12864-022-08445-4

**Published:** 2022-03-16

**Authors:** Yanglei Jia, Xiao Liu

**Affiliations:** Fishery College of Zhejiang Ocean University, Zhoushan, Zhejiang China

**Keywords:** Oysters, Adaptation, Aquaporin, Tandem duplication, Functional differentiation

## Abstract

**Background:**

The diversified aquaporin (AQP) family that was derived from gene duplication and subsequent functional differentiation play critical roles in multiple physiological processes and in adaptation to the dynamic environments during the evolutionary process. Oysters are a group of bivalve fauna in Mollusca that were widely distributed around the world and show extraordinary adaptation to harsh environments. However, knowledge is lacking with the diversity and evolution of the AQP family in oysters, even in molluscs.

**Results:**

Here, we performed a comprehensive analysis of the AQP family in three geographical isolated oyster species that are native to different environments. Genome distribution and phylogenetic analysis revealed that the expansion of the AQP family in oysters were attributed to tandem duplication. Synteny analysis indicated that large-scale inversions lead to the independent duplication or deletion of the AQPs after speciation. As a consequence, these independent duplication events contributed to the diversification of the AQP family in different oysters. Pore pattern analysis suggested that the duplicated AQPs in oysters were highly diversified in inner surface profiles, implying the subsequent functional differentiation. The comparison conducted based on the transcriptome data demonstrated that the functional differentiated AQP family members in oysters may play critical roles in maintaining the balance between the stationary homeostasis and dynamic environments.

**Conclusions:**

Our observation provides evidence for the correlation between the duplicated and functional differentiated AQP family and the adaptation to stationary life under dynamic environments in oysters. Additionally, it also broadens our knowledge of the evolution of AQP family in molluscs.

**Supplementary Information:**

The online version contains supplementary material available at 10.1186/s12864-022-08445-4.

## Background

Oysters are a group of marine bivalve molluscs that belong to the family Ostreidae. As one of the most productive molluscs, oysters are well known for the established commercial importance in the fisheries and aquaculture industries [1]. Besides, oysters also serve as keystone species in the marine ecosystem and play important roles in water filtration and reef systems [2]. Climatic variation and crustal movements promote the expansion, extinction and distribution of oysters around the world’s oceans [1]. Moreover, the extant sessile oysters in the world are mainly distributed in the intertidal zone of the coast. As a consequence, oysters have evolved extraordinary resilience to the harsh environmental stress in the intertidal coast, especially for the dynamic environments such as temperature, salinity and air exposure [3]. Fluctuations of the environmental factors have been recognized as a critical force in the geographic separation of oysters [1]. Meanwhile, the diversity of genetics in oysters were also shaped for adaptation to the changing environments during the process of evolution [4, 5]. Besides, the variations within different oysters in shape, color and even the taste of its meat are significantly influenced by the surroundings and the method of cultivation. In this context, oysters represent an attractive model for studying the detailed mechanism in stress adaptation. Genetic and phenotypic diversity derived from genomic structural variation play a critical role in adaptation to the dynamic environments during the process of evolution [6, 7]. Understanding the genome diversity of oysters at species level is central to revealing the adaptation mechanism to the varietal environments.

Generally, aquaporin (AQP) was a highly expanded and diversified gene family that closely related to environmental adaptation in broader phyla during the process of evolution [8]. AQP is a group of conserved channel proteins that mediate the selective permeation of water molecules and some other solutes like glycerol and ammonia [9, 10]. As an important channel family that facilitates the permeation of small molecules and regulates fluid homeostasis in various environments, AQPs are present in almost all living organisms. The diversified AQPs were divided into four clades corresponding to classical AQPs, aquaglyceroporins (Glps), AQP8 and unorthodox AQPs, with each representing a subfamily and performing distinct biofunctions [8, 9, 11, 12]. Gene duplication and subsequent functional diversification are the molecular basis for the evolution and differentiation of the AQP family [13, 14]. As a consequence, the total number of the AQP genes in multicellular animals was highly diversified when compared with that in prokaryotes and protists [9]. For instance, genome wide duplication (WGD) events that occurred in the common ancestor of the vertebrates are the basis for the diversification of the AQP family in tetrapods [10, 15]. Noteworthily, a region that is named aromatic/arginine (Ar/R) region and built by four separated amino acids in the protein sequence functioned as a selective filter [16]. The diversification of the amino acids in this region was regarded as the basis for the functional differentiation. From the physiological perspective, the diversified AQP family plays critical roles in osmotic pressure regulation, nutrient metabolism and other physiological processes [17, 18]. From the evolutionary perspective, the diversification and subsequent functional differentiation of the AQP family has biological significance for different species in adaptation to the environment changes [10, 19, 20].

Intriguingly, as the second largest phylum in animals and accounting for 7% of all living animals on earth, molluscs inhabit extensive variable environments including terrestrial, marine and freshwater habitats [21, 22]. Access to water is the most important environmental factor for living organisms, especially for the aquatic species. In this context, the composition pattern of the AQP family was also highly diversified and showed low collinearity in different species of molluscs [23]. In spite of their importance, molluscan aquaporins have not been thoroughly investigated. Up to now, rare studies have been performed on the molluscan AQP families [23–25]. Moreover, the duplication and deletion of the AQP family members during the process of evolution were rarely reported in molluscs. Unexpectedly, some AQP orthologs that show untypical characteristics in the Ar/R region were detected in molluscs [23]. As a selection filter, the composition pattern of amino acids in the Ar/R region is one of the major bases for the differentiation between different AQP subfamilies. Similar phenomenon also appeared in holometabolan insects to compensate for the absence of Glp subfamily [26]. While the duplication pattern of the atypical AQPs were independently specialized in molluscs and insects [25]. Except that, little is known about the origin, transport properties and even the evolutionary forces for the atypical AQPs in molluscs.

Considering that the widespread oysters inhabit extremely diverted environments, the composition pattern of the AQP family in these species was rarely reported. In order to understand the enhanced tolerance to the varietal environmental conditions in oysters, we have now conducted the duplication and deletion of the AQP family in *Crassostrea* based on the genome wide comparison. Three widely harvested oysters including the *Crassostrea gigas* (Cgi, Pacific oyster) that natively originated along the coasts of northeastern Asia [27], the *Crassostrea hongkongensis* (Chk, Hong Kong oyster) that cultivated surrounding the coastal of the Pearl River Delta in southern China [7] and the *Crassostrea virginica* (Cvi, Eastern oyster) that distribute from Gulf of Mexico to the Gulf of St. Lawrence along the Eastern Atlantic coast [28] were selected to perform this analysis. It is notable that the three selected oysters are distributed in different geographical environments and show long evolutionary distances [1]. Moreover, the published good reference genomes for these three oysters provide the feasibility to analyze the evolution of the AQP families. Comparative analyses in present research were carried out to provide novel insight to understand the relationship between the diversified molluscs AQP family and the corresponding habit environment in evolutionary perspective.

## Results

### Massive expansion of the AQP genes in oysters

**Fig. 1:**
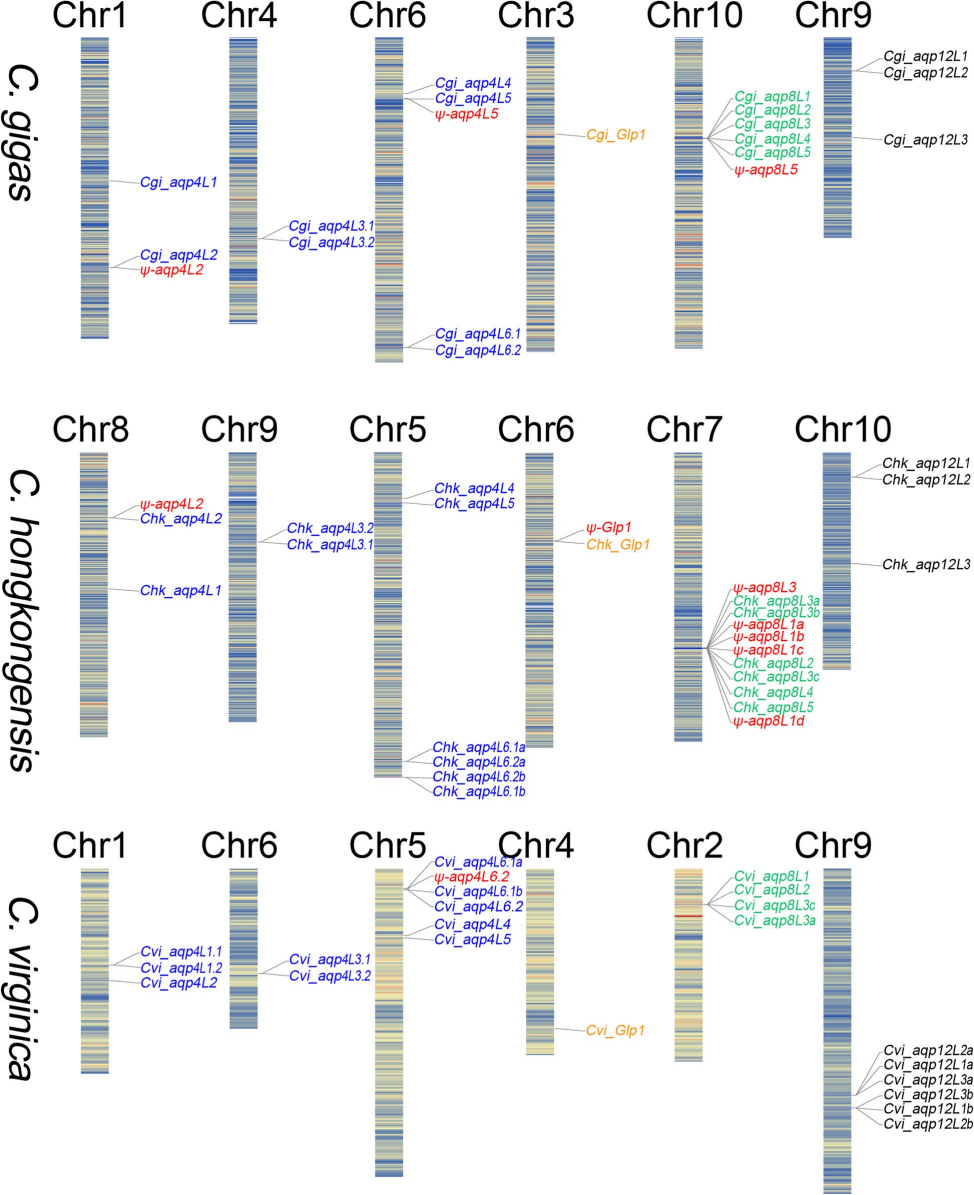
Chromosome distribution of the AQP family members in different oysters. The complete set of the AQP genes were de novo identified based on the genome assemblies. The tandem duplicated orthologs were separated by appending a “.1” and “.2” at the end of the names. The pseudogenized AQPs were appended with a “ψ” at the front of the gene names and marked with red. The members clustered into the classical AQP subfamily were marked with blue. The members clustered into the Glp subfamily were marked with orange. The members clustered into the AQP8 subfamily were marked with green. The members clustered into the unorthodox AQP subfamily were marked with black

**Fig. 2:**
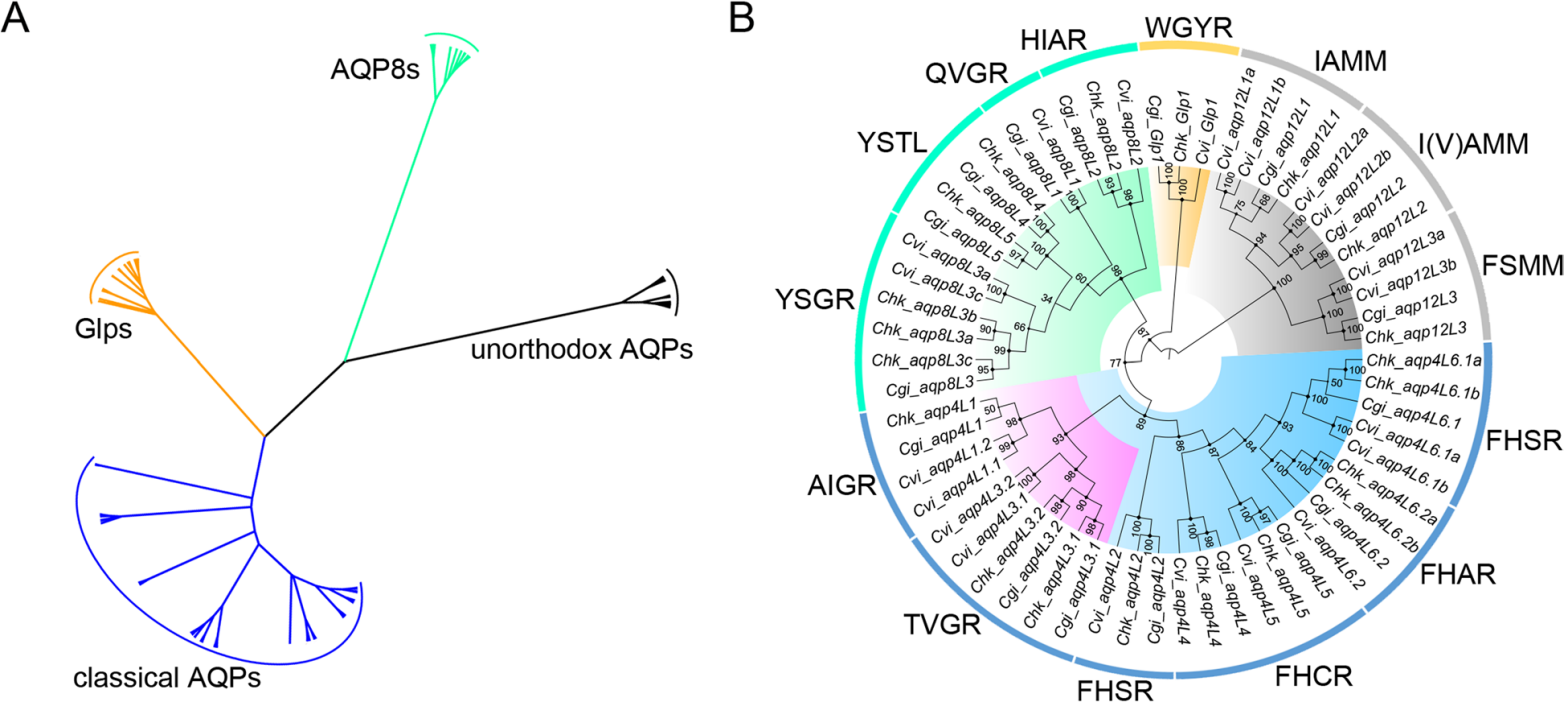
Phylogenetic analysis of the AQPs. **A** Phylogenetic analysis of the complete set of the AQP family members in different oysters and combined with that collected from the other species. The four distinct clusters represent the four subfamilies in AQP family. **B** Phylogenetic analysis of the AQP family members in three oyster species. The composition pattern of the amino acids in the Ar/R region that independently marked around the dendrogram were identified based on the alignments of the protein sequences. The separated subfamilies were highlighted in different colors. Besides, the members with atypical Ar/R region in the classical AQP subfamily were highlighted in pink

Unexpectedly, the composition pattern of the AQP family in oyster species were highly expanded (Fig. 1). Besides, several pseudogenized AQP genes were also detected in these three species. It is clear that the complete set of the AQP family members in oysters were distributed across 6 of the 10 chromosomes (Fig. 1). Moreover, the distribution of the AQP genes in different oyster species were also highly conserved as expected. Remarkably, phylogenetic analyses revealed that the complete set of the AQP genes were clearly categorized into four distinct clusters, representing the four independent subfamilies (Fig. 2A). It is noteworthy that all of the four AQP subfamilies are presented in oyster species. Additionally, the members that are localized at the consistent region of the chromosome between different oysters also cluster into the same subbranches (Fig. 2B). Besides, some members that adjacent localized at the same chromosome were also clustered together.

It is obvious that about half of the AQP family members were clustered into the classical AQP subfamily (Fig. 2B). However, most of the members in this subfamily were separately distributed in different chromosomes (Fig. 1). Additionally, the identity between the separately distributed orthologs in the same subfamily were also much lower than that between the tandem duplicated orthologs (Additional file 1: Figure S1A). In this context, the complete set of the AQP genes in this subfamily could be divided into six orthologs based on the phylogenetic analysis (Fig. 1 and 2B). Noticeably, tandem duplications have occurred on majority of the separately distributed orthologs in the classical AQP subfamily.

The most prominent feature of the AQP family in oysters was the significant expansion of the members in the AQP8 subfamily (Fig. 2B). In summary, five orthologs that clustered into the AQP8 subfamily have been detected in oysters. Similarly, tandem duplication led to the highly diversification of the AQP8 subfamily in oysters. However, the members that clustered into the AQP8 subfamily were also highly varied between different oysters (Fig. 1). Except for the subfamilies that mentioned above, tandem duplication was also detected in the unorthodox AQP subfamily (Fig. 1). Unexpectedly, only one member was clustered into the Glp subfamily (Fig. 1 and 2B). Similar features were not familiar in most multicellular organisms.

## Duplication and deletion of the AQPs in oysters

Synteny analysis revealed that the arrangement of the genes in chromosomes showed high collinearity between Pacific oyster and Hong Kong oyster. In contrast, large scale inversions and even chromosome breakage and refusion were detected in eastern oyster when compared with the other two oysters (Additional file 2: Figure S2A). However, the region that encoding the AQP orthologs were still highly conserved in these oysters based on the synteny analysis (Additional file 2: Figure S2B). The variety of AQP genes in these three oyster species suggests that some orthologs may be independent duplicated or deleted during the process of evolution.

**Fig. 3:**
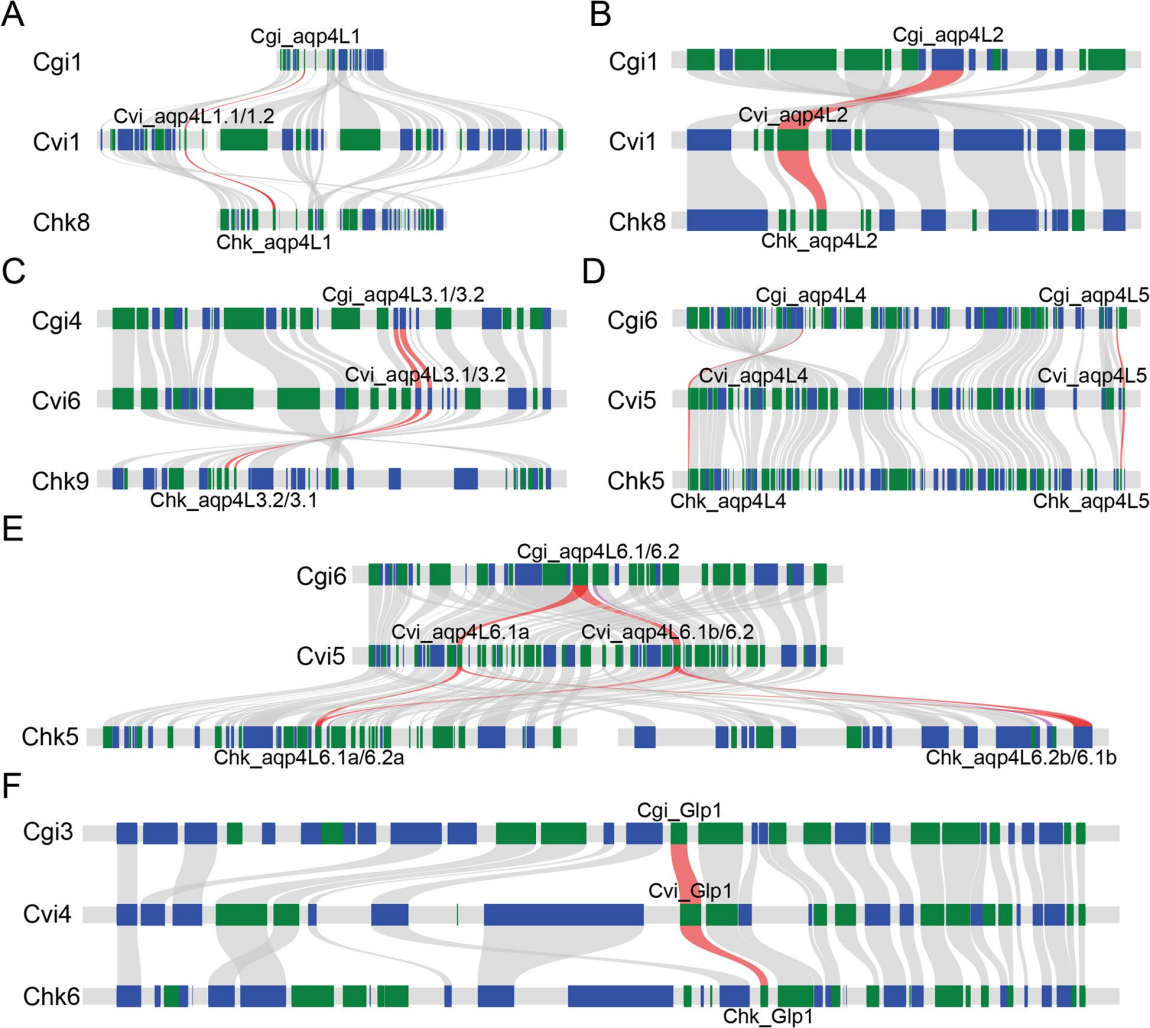
Duplication and deletion of the orthologs in classical AQP and Glp subfamilies in oysters. **A** Synteny analysis of region that encoding the Aqp4L1 ortholog. **B** Synteny analysis of the region that encoding the Aqp4L2 ortholog. **C** Synteny analysis of the region that encoding the Aqp4L3 ortholog. **D** Synteny analysis of the region that encoding the Aqp4L4 and -4L5 orthologs. **E** Synteny analysis of the region that encoding the Aqp4L6 ortholog. **F** Synteny analysis of the region that encoding the Glp1 ortholog

It is clear that the Aqp4L1 ortholog was further duplicated in eastern oyster and no trace of deletion has been detected in the other two species (Fig. 3A). Synteny analysis revealed that this ortholog was localized at the edge of the inverted region in eastern oyster (Fig. 3A). In contrast, the consistent regions show high collinearity in Pacific oyster and Hong Kong oyster based on the synteny analysis. The inversion event that independently occurred in this region may contribute to the duplication of the Aqp4L1 orthologs in eastern oyster. Additionally, the high identity (higher than 90% including intron and exon segments) between the duplicated Aqp4L1 orthologs in eastern oyster (Additional file 1: Figure S1A) revealed the independent tandem duplication events that have lately occurred after the speciation event.

Synteny analysis revealed that the arrangement of the genes that distributed around the region encoding Aqp4L2 ortholog show high collinearity in different oysters (Fig. 3B). Remarkably, a pseudogene that shows high conservation with Aqp4L2 has been detected in both Pacific oyster and Hong Kong oyster (Fig. 1). Blast analysis revealed that several sites with nucleotide acids insertion or deletion that leads to the shift on the reading frame have been detected in the pseudogenized Aqp4L2 orthologs (Additional file 3: Figure S3A). Moreover, some exons in these pseudogenes were also absent based on the sequence alignment. It is clear that the duplicated Aqp4L2 orthologs were gradually pseudogenized in these two oyster species during the process of evolution. Nevertheless, similar duplication and subsequent pseudogenization were not detected in eastern oyster. Additionally, the reserved segments of the pseudogenized Aqp4L2 in these two oysters show high conservation with the retained ortholog. These data revealed that the duplication event was independently occurred in the last common ancestor of Pacific oyster and Hong Kong oyster after speciation.

Remarkably, tandem duplication events also occurred in all of the three oyster species in the region that encoding Aqp4L3 (Fig. 3C). Strikingly, the extensive conservation of the duplicated Aqp4L3 suggests a recent duplication event in oysters (Additional file 1: Figure S1B). Unexpectedly, the identity of the corresponding Aqp4L3 orthologs between eastern oyster and the other two oysters was lower than that between the two duplicated orthologs in eastern oyster (Additional file 1: Figure S1B). In contrast, the identity between the consistent copy of Aqp4L3 in Pacific oyster and Hong Kong oyster were higher when compared with that between the two duplicated orthologs in the independent species (Additional file 1: Figure S1B). Moreover, phylogenetic analysis also revealed that the duplicated Aqp4L3 orthologs in eastern oyster were clustered together rather than independently clustered with the corresponding orthologs in the other oyster species (Fig. 2B). These data revealed that the Aqp4L3 orthologs in eastern oyster and the other two species may independently duplicated after the speciation event. Additionally, the differentiation between Pacific oyster and Hong Kong oyster was later than the tandem duplication events.

The next tandem duplicated orthologs in the classical AQP subfamily were named Aqp4L6. It is clear that the duplicated Aqp4L6 orthologs in oyster could be distinctly separated into two groups based on the identities between each other (Additional file 1: Figure S1C). Similar duplication event was also detected in Sydney rock oyster *Saccostrea glomerata*, a species that clustered into Saccostrea (data not shown). These data indicated the tandem duplication that occurred in this region was a common ancient event in Ostreidae. The striking characteristic around the region encoding these orthologs is the independent large-scale tandem duplication events that occurred in eastern oyster (about 320 kb) and Hong Kong oyster (about 240 kb) (Fig. 3E). Moreover, inner-chromosome rearrangements also occurred after the large-scale tandem duplication in Hong Kong oyster (Fig. 3E). Besides, blast analysis indicated that one nucleic acid deletion was occurred in one of the duplicated Aqp4L6.2 ortholog and lead to the pseudogenization in eastern oyster (Additional file 3: Figure S3C) after the large-scale tandem duplication.

Apart from the orthologs mentioned above, the remaining two orthologs (Aqp4L4 and Aqp4L5) in the classical AQP subfamily were separately localized in the same chromosome (Fig. 3D). The average distance between these two orthologs in the chromosome was more than 800 kb in oysters. Moreover, synteny analysis revealed that several inversion events were also detected around these two orthologs. Besides, these two orthologs were all coincidentally localized at the edge of inverted regions (Fig. 3D). These data give us a clue that the separate distribution of Aqp4L4 and -4L5 may be attributed to the ancient subsequent multiple inversion events that occurred after the tandem duplication between them. It should be noted a pseudogene that was not adjacent localized with Aqp4L5 but appeared downstream (100 kb) of it was detected in Pacific oyster. While only the first exon segment was fully retained in the pseudogenized ortholog (Additional file 3: Figure S3B). Besides, the intron sequences on both sides of the first exon also show high conservation. These data suggested that duplication and subsequent arrangement may occur around the region in this oyster. Except that, no duplication and deletion have been detected around this region in the other oysters. Similar phenomena also occurred upstream of the region that encoding the single Glp ortholog in Hong Kong oyster (Additional file 4: Figure S4A). Remarkably, except for the last two exon segments, the other exons were all absent in the pseudogenized Glp sequence. It is clear that the regional inversion events that occurred in the upstream segments of Glp1 in oyster may lead to the duplication and separative localization of the consistent AQP ortholog in Hong Kong oysters when compared with the others (Fig. 3F).

**Fig. 4:**
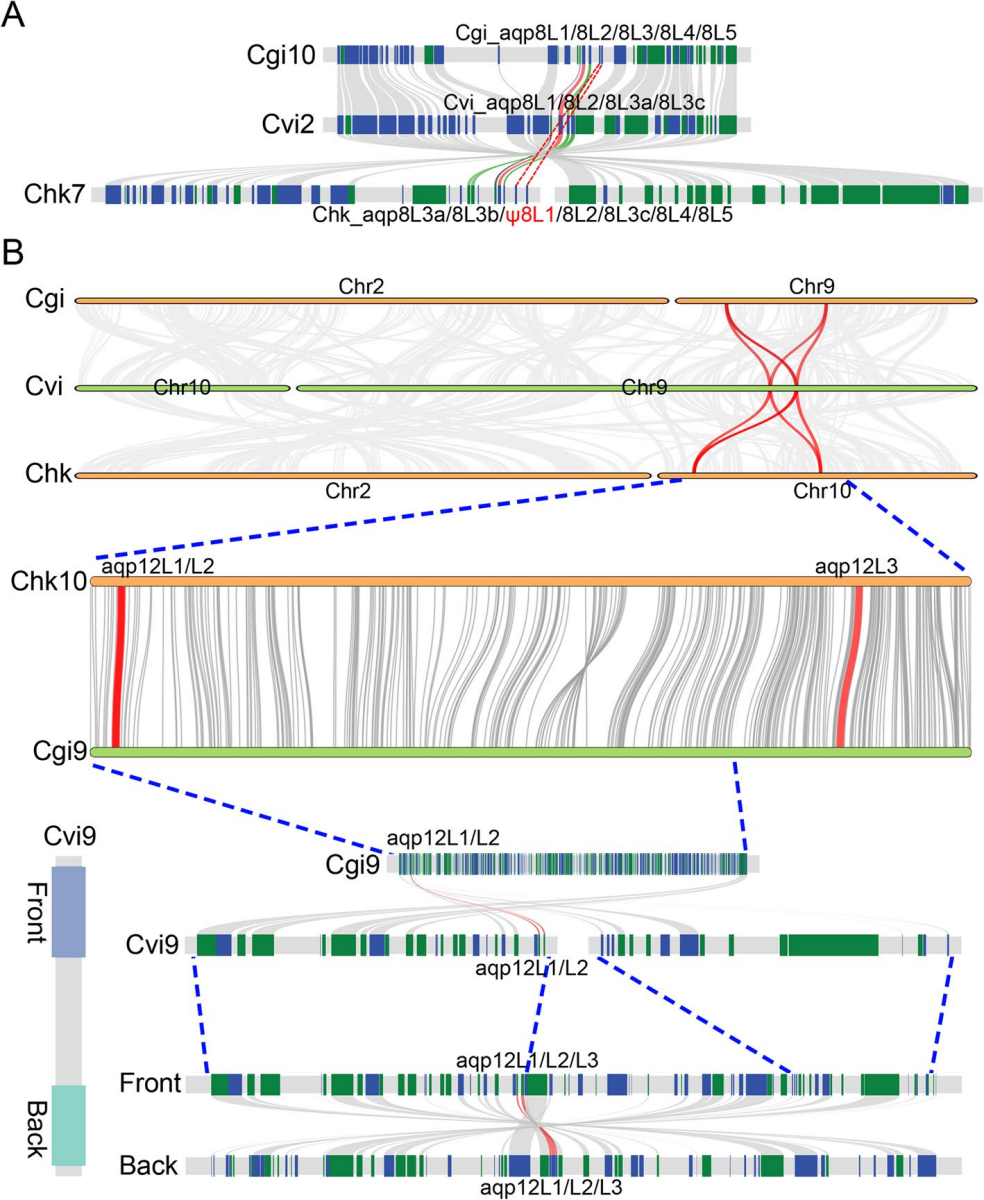
Duplication and deletion of the orthologs in AQP8 and unorthodox AQP subfamilies in oysters. **A** Synteny analysis of region that encoding the AQP8 orthologs. **B** Synteny analysis of the region that encoding the unorthodox AQP ortholog

As mentioned above, the AQP8 subfamily were extensive duplicated and five orthologs have been detected in oysters. It is clear that the tandem duplicated AQP8 orthologs could be divided into four subclades. The orthologs of Aqp8L4 and -8L5 showed extensive conservation when compared with that between the others (Additional file 1: Figure S1D). Unexpectedly, both of these two duplicated orthologs were absented in eastern oyster (Fig. 4A). Additionally, similar duplicated AQP orthologs were also not detected in the *Saccostrea glomerata*, a species that clustered into Saccostrea (data not shown). These data revealed that the arising of these two duplicated orthologs may have occurred after the speciation of the common ancestor for Pacific oyster and Hong Kong oyster.

Intriguingly, the Aqp8L1 ortholog was abundant duplicated in Hong Kong oyster independently (Fig. 1). However, all of them were gradually pseudogenized during the process of evolution (Additional file 4: Figure S4B). It should be noted that the whole exon segments were completely reserved in the ψChk_aqp8L1c and show high identity to the corresponding ortholog in Pacific oyster. Unexpectedly, a long-block insertion was identified in the third exon based on the blast analysis (Additional file 4: Figure S4C). While no splicing sites were detected at the terminal of the inserted segment. Remarkably, similar segments that show high conservation to the insertion in both identity and length were widely distributed in the genome assemble (Additional file 4: Figure S4D). Besides, a single nucleotide deletion that led to the reading frame shift was also identified at the end of the fourth exon segment (Additional file 4: Figure S4B). In this context, no functional Aqp8L1 ortholog has been detected in Hong Kong oyster.

Remarkably, Aqp8L3 was another subsequently duplicated orthologs in eastern oyster and Hong Kong oyster. While similar duplication events were not detected in Pacific oyster. Unexpectedly, the orthologs that belong to Aqp8L3 were also highly diversified based on the phylogeny analysis (Fig. 2B and Additional file 1: Figure S1D). It should be noted that the identity between the two duplicated Aqp8L3 orthologs in eastern oyster show higher profiles when compared with that in the other two oysters (Additional file 1: Figure S1D). Besides, the three duplicated Aqp8L3 orthologs in Hong Kong oyster were not adjacently localized in the chromosome. It is clear that some inversion events that lead to the inner chromosome arrangements have occurred in this region during the process of evolution (Fig. 4A). Additionally, the duplication between Aqp8L3a and -8L3b may have occurred after the inversion events in the Hong Kong oyster. These data suggested that the duplication of Aqp8L3 orthologs in various oyster species were not attributed to the common events that occurred in the ancient oysters. Instead, the tandem duplication of Aqp8L3 orthologs in different oysters occurred independently after the speciation event.

The unorthodox AQP subfamily was special for its high diversity and low identity in sequence between the members. In summary, there are three unorthodox AQP orthologs in oysters. Similar to the members in the other subfamilies, tandem duplication also contributed to the abundance of the unorthodox AQP subfamily in oysters (Fig. 1). Remarkably, the identity between Aqp12L3 and the rest two orthologs was much lower than that between Aqp12L1 and -12L2 (Additional file 1: Figure S1E). These data revealed the tandem duplication that occurred between the Aqp12L1 and -12L2 orthologs was the latest duplication event in this region. However, phylogenetic analyses revealed that all of the duplicated unorthodox AQP orthologs in this region were derived from the common ancestor of oysters. It is noteworthy that the ortholog of Aqp12L3 were separately localized with the other two unorthodox AQP orthologs in Pacific oyster and Hong Kong oyster, which is clearly distinguished from that in eastern oyster (Fig. 1). Remarkably, large-scale inversion and rearrangement events that occurred after the differentiation of the common ancestor for these two species lead to the separation of the duplicated unorthodox AQP orthologs (Fig. 4B). Additionally, breakage and random fusion of the chromothripsis were also detected in the consistent region in eastern oyster based on the synteny analysis (Fig. 4B). Besides, large-scale tandem duplication (about 150 kb) that leads to the abundance of unorthodox AQP subfamily even occurred in eastern oyster. However, the consistent chromosomes in Pacific oyster and Hong Kong oyster show high collinearity.

## Gene structure conservation of the AQPs in oysters

**Fig. 5:**
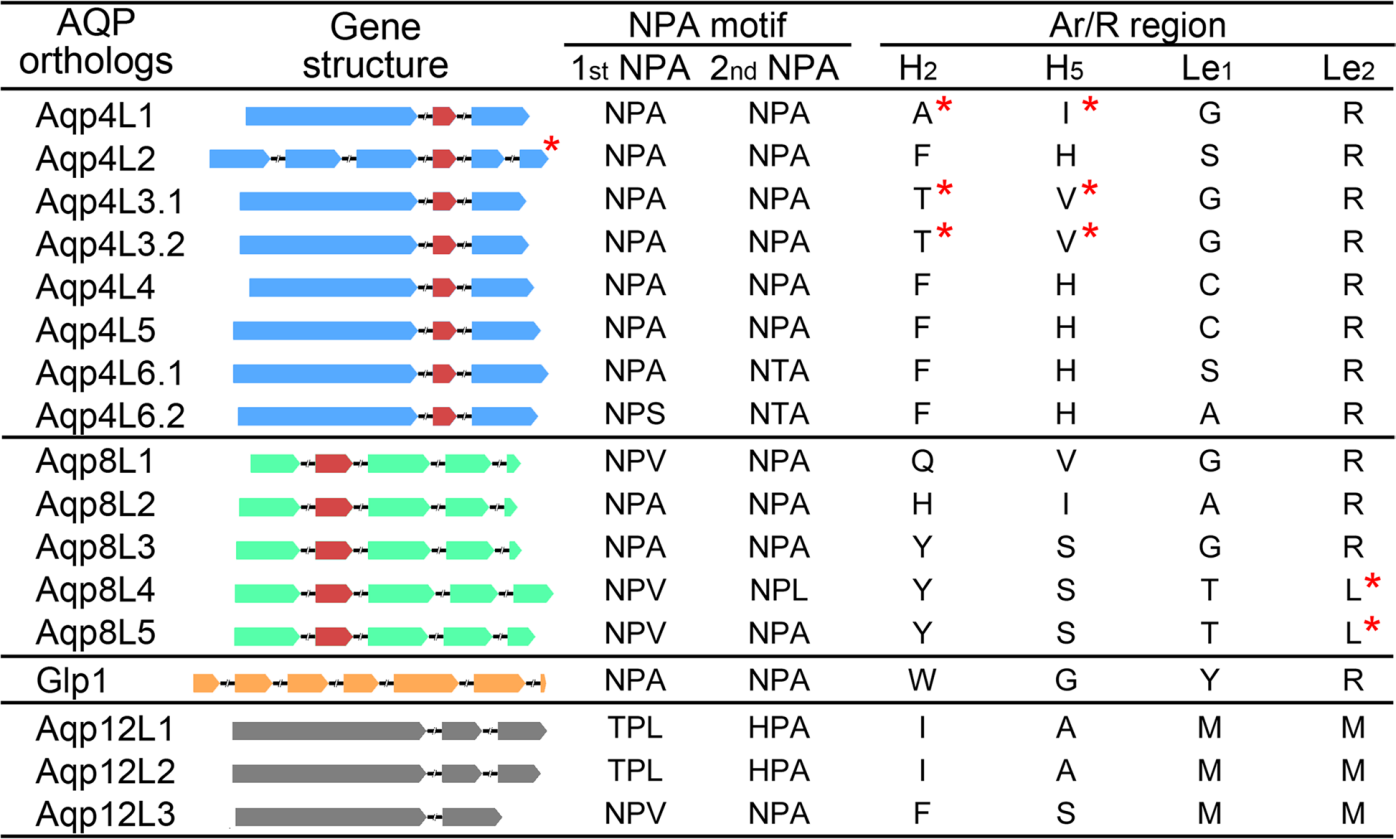
Gene structure of the AQP orthologs in oysters. The wedge squares in different colors represent the exon regions of the AQPs in different subfamilies. The introns were showed with the shrinked lines in same length. The conserved exons in different subfamilies were showed with the red wedge squares. The amino acids in the NPA motif and Ar/R region were summarized based on the protein sequence alignments. The structure or the amino acids that marked with the red “*” represent the corresponding ortholog contain atypical feature of when compared with the other members in the same subfamily

The composition pattern of the exon/intron structure for different AQP orthologs was visualized based on the annotation information. Gene structure analysis indicated that the members clustered into the same subfamily showed high conservation when compared with the others, although most of them were separately distributed in different chromosomes (Fig. 5). It should be noted that except Aqp4L2, all the other orthologs in the classical AQP subfamily contain the similar exon composition pattern. It is clear that the first long exon that was conserved in the classical AQP subfamily was fragmented into three short exons by the inserted introns in Aqp4L2. Similarly, the conserved exon-3 was also separated into two parts in this ortholog. These data suggested the difference in evolutionary origins between Aqp4L2 and the others. Interestingly, a highly conserved exon composed of 81 nucleotide acids and encoding 27 amino acids was detected in the classical AQP subfamily members. Coincidentally, the conserved exon was consistent with the segments encoding the second NPA motif in this subfamily. Except that, the difference of the rest exon segments in length reflected the variable of the cytoplasmic extensions in the N- and C-terminal, which also function as a switch to control the permeability of the channel [29].

Strikingly, similar phenomena were also detected in the AQP8 subfamily. It is obvious that the second exon in the AQP8 orthologs, which was composed of 127 nucleotide acids and encoding 43 amino acids, showed high conservation when compared with the others (Fig. 5). Except that, the rest exons were variable in length between each orthologs based on the alignment of these orthologs using the amino acid sequences (Additional file 5: Figure S5). In contrast to that in the classical AQP subfamily, the conserved exon segments in AQP8 subfamily were consistent with the region that encoding the first NPA motif. Unexpectedly, the duplicated Aqp8L3 orthologs in Hong Kong oyster were also diversified in exon length when compared with that in other oyster species (Additional file 5: Figure S5). The diversification of the exons in length may lead to the variety of the inner surface pattern of these orthologs in the AQP8 subfamily.

It should be noted that the member in the Glp subfamily contains the most exons when compared with the others (Fig. 5). Additionally, no exons in the Glp ortholog show any conservation to that in the other subfamilies. Unorthodox AQP is another tandem duplicated subfamily in oysters. As the subfamily was characterized by the high diversity and low identity in sequence, the gene structure of these members was also distinguished from the members in the other subfamilies (Fig. 5). It should be noted that the Aqp12L3 ortholog was also distinguished from the other two members in gene structure, which is consistent with the conclusion of phylogenetic analysis. It is obvious that the exon segment that corresponds to the second exon in Aqp12L3 was separated into two parts in the other two members before the duplication events occurred between Aqp12L1 and -12L2.

## Structure and filter region diversity of the AQPs in oysters

Composition of the amino acid residues in the two NPA motifs and the Ar/R region are regarded as the basis for the functional differentiation of AQPs in different subfamilies. In this context, the amino acids in these two regions were summarized based on the alignments of the protein sequences (Fig. 5). It is clear that except for the members in the unorthodox AQP subfamily, the composition of amino acids in the NPA motifs were highly conserved in these AQP orthologs despite the difference of subfamilies clustered in oysters. However, the amino acids in the Ar/R region were all extensively diversified in different subfamilies (Fig. 5).

**Fig. 6:**
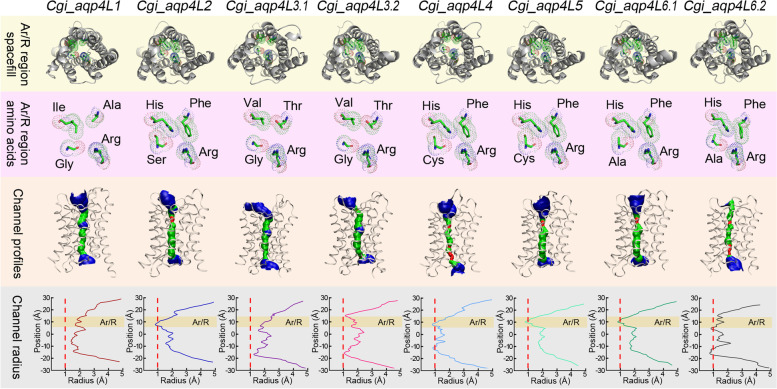
Apertures and architecture analysis of the inner surface for the members in the classical AQP subfamily in oysters. The graphs in the first line represent the individual structure for each member from the non-cytoplasm side. The amino acids in the Ar/R region were highlighted by elements. The graphs in the second line represent the corresponding partial enlarged view of the amino acids in the Ar/R region. The graphs in the third line represent the independent inner surface profiles of the channel along the z-axis. The regions colored by red represent the radius was narrower than 1 Å. The regions colored by green represent the radius was varied from 1 to 2 Å. The radius of the regions that larger than 2 Å were colored by blue. The graphs in the fourth line represent the individual channel radius curves along the z-axis

Generally, the first two amino acids in the Ar/R region in the classical AQP subfamily members were extensively conserved and typically appeared as Phe and His. Unexpectedly, the amino acids in the consistent sites in Aqp4L1 and -4L3 orthologs showed untypical patterns. The conserved Phe and His that contained a large ring structure in the side chain were replaced by Ala and Ile respectively in Aqp4L1 (Fig. 5). Calculation of the pore inner-surface profiles suggested that the replacement in these sites definitely enlarged the diameter of these orthologs in the Ar/R region (Fig. 6). Besides, the conserved Phe and His were replaced by Thr and Val respectively in Aqp4L3 orthologs. Similar to that in Aqp4L1, these replacements that occurred in Aqp4L3 also enlarged the diameter of the channel around the Ar/R region (Fig. 6). As mentioned above, the ortholog of Aqp4L3 in oysters were tandemly duplicated in different oysters. Additionally, the composition of amino acids in the Ar/R region of the duplicated Aqp4L3 orthologs was even highly conserved. However, the pore pattern of these duplicated orthologs were eventually differentiated based on the construction of the inner surface (Fig. 6). These data suggested that except for the amino acids in the Ar/R region, some other selective permeation determination sites were differentiated after the duplication of this ortholog.

Aqp4L6 was another duplicated ortholog in the classical AQP subfamily in oysters. It should be noted that both of the duplicated Aqp4L6 orthologs in oysters contain the typical composition pattern of amino acids in the Ar/R region (Fig. 5). Remarkably, the conserved Phe and His residues at the first two sites in the Ar/R region were all reserved in these duplicated orthologs. However, the pore pattern of these channels were differentiated throughout between the duplicated Aqp4L6 orthologs (Fig. 6). It is clear that the diameter of the channel below the Ar/R region was also extremely narrowed in Aqp4L6.2 when compared with that in the consistent region in Aqp4L6.1 (Fig. 6). These data proposed that the function of these tandem duplicated AQP orthologs were highly differentiated subsequently.

Similar phenomena also appeared in the ortholog of Aqp4L4 and -4L5 (Fig. 3D and 5). It should be noted that these two separately distributed members share the same composition pattern of the amino acids in the Ar/R region (Fig. 5). These data suggested that the separately distributed two AQP orthologs may share the same evolutionary origin in the ancient ancestor. Tandem duplication and subsequent large-scale inner chromosome inversions lead to the abundance and separation of these two orthologs. Notably, the conserved Phe and His in the Ar/R region significantly compressed the diameter of the channel around this part. However, the pore pattern of Aqp4L4 was differentiated throughout when compared with that in Aqp4L5 (Fig. 6). Similarly, the diameter of the channel below the Ar/R region were also extremely narrowed. As expected, functional differentiation also occurred between these two AQP orthologs.

**Fig. 7:**
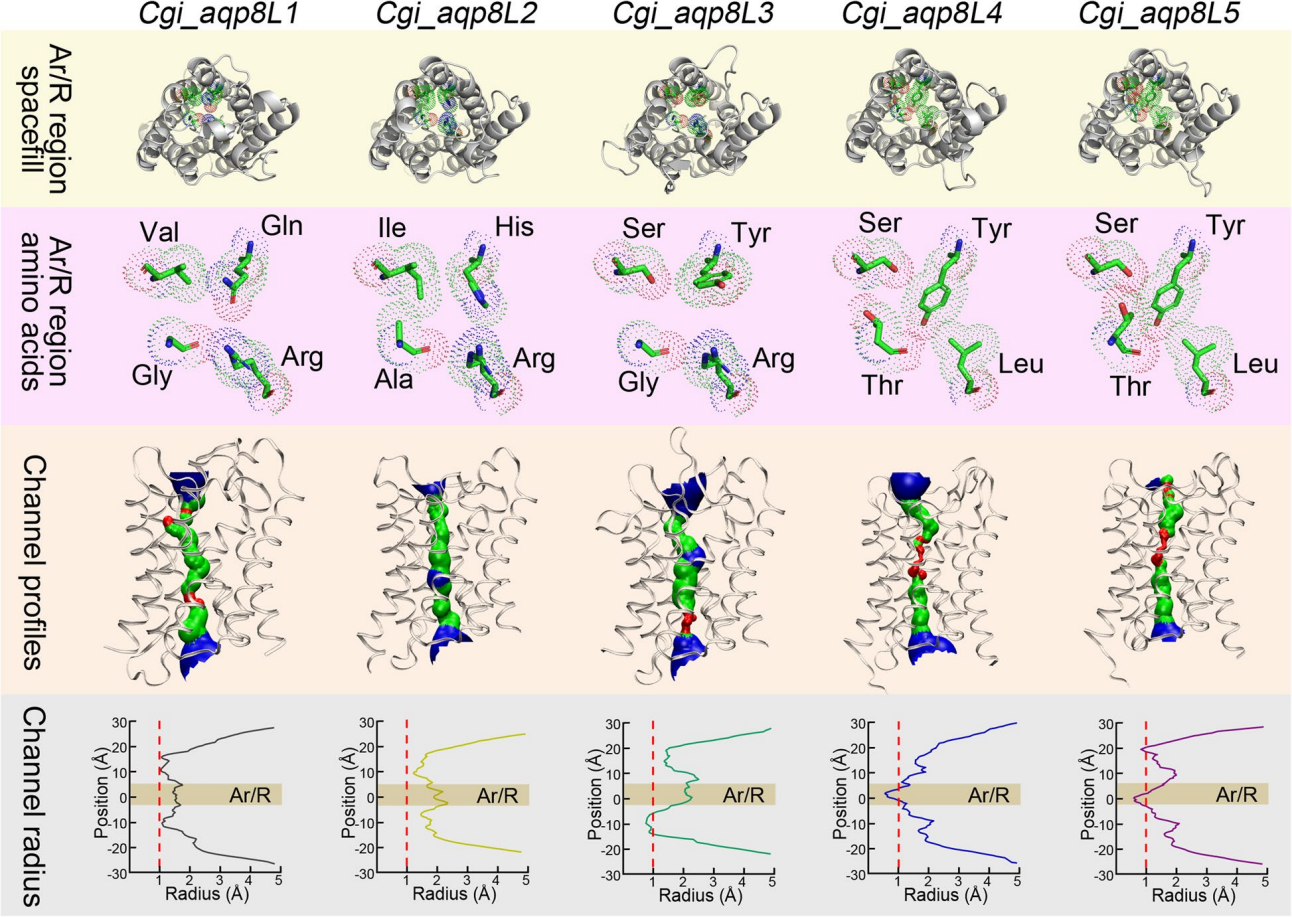
Apertures and architecture analysis of the inner surface for tandemly duplicated members in the AQP8 subfamily in oysters. The graphs in the first line represent the individual structure for each member from the non-cytoplasm side. The amino acids in the Ar/R region were highlighted by elements. The graphs in the second line represent the corresponding partial enlarged view of the amino acids in the Ar/R region. The graphs in the third line represent the independent inner surface profiles of the channel along the z-axis. The regions colored by red represent the radius was narrower than 1 Å. The regions colored by green represent the radius was varied from 1 to 2 Å. The radius of the regions that larger than 2 Å were colored by blue. The graphs in the fourth line represent the individual channel radius curves along the z-axis

As described above, AQP8 was the extensively duplicated subfamily in oysters during the process of evolution (Fig. 1). Remarkably, the composition pattern of the amino acids in the Ar/R region, which function as a selection filter, was also extremely diversified in these duplicated orthologs in the AQP8 subfamily (Fig. 5). Generally, the amino acid in the first site in the Ar/R region was highly conserved and typically appeared as His in AQP8 subfamily members. Thus, the conserved His at this position has been regarded as a common feature among the AQP8s both in plants and animals. Interestingly, only the Aqp8L2 ortholog in this subfamily shows the typical composition pattern of the amino acids in the Ar/R region in oysters (Fig. 5). While in the other orthologs in this subfamily, the positive-charged His in these consistent sites were replaced by uncharged Tyr or Gln. It should be noted that the appearance of Tyr in this position in Aqp8L4 and -8L5 orthologs significantly compressed the aperture of the channel. In contrast, similar phenomenon did not appear in the Aqp8L3 ortholog, although the conserved amino acid replacement was occurred in this consistent position. The orientation of the benzene ring structure on the inner surface may have an important effect on the diameter of the channel. Except that, the conserved positive charged Arg in the Le2 site in the Ar/R region were all replaced by Leu, an amino acid with a hydrophobic side chain, in the Aqp8L4 and -8L5 orthologs (Fig. 5 and 7). In this condition, the inner surface of these channels show extreme hydrophobicity in the Ar/R region. It should be noted that the Aqp8L1 also shows an untypical composition pattern of the amino acids in the Ar/R region, although the diameter of the channel in the consistent region was not obviously compressed. Nevertheless, the characteristic of the inner surface may be changed due to these replacements (Fig. 7). It is obvious that the diversification of the amino acids in the Ar/R region are the basis for the functional differentiation after the duplication events in the AQP8 subfamily in oysters. However, the detailed selection mechanism of these duplicated orthologs still needs further investigation.

As the Glp subfamily in oysters was highly contracted and the retained one member also showed typical composition pattern of the amino acids in the Ar/R region, the detailed pattern of the inner surface was not performed in this study. Besides, considering the extensive diversification of the members in the unorthodox AQP subfamily, while no available template could be used to perform the construction of pore pattern for the members in this subfamily. In this context, the influence of amino acids in the Ar/R region on the diameter of the channel was also not performed in the present research. However, the conserved Arg on the last site in these orthologs were all replaced by Met in the Ar/R region, suggesting the higher functional differentiation of members in the unorthodox AQP subfamily.

## Functional diversity of AQP family members in oysters

As a family of channel proteins that facilitated the trans membranes of some solutes, AQPs regulate a variety of biological processes and play critical roles in a wide range of physiological functions. The transcriptome data including different developmental stages and different adult organs revealed the diversified expression pattern of the complete AQP family genes in Pacific oyster, especially for the duplicated orthologs (Additional file 6: Figure S6). Overall, the diversified expression patterns of AQP family members are gradually appeared combined with the process of embryo development. Besides, some AQP family members were expressed in a stage specific manner. While in adult organs, some of the abundant AQP family members are tissue-specific expressed, which may reflect the diversification of the duplicated members in specific regulation or functional compartmentalization. Additionally, some of the tandemly duplicated orthologs were also differentiated expressed based on these analyses. Taking together, these data suggested the complex biofunction of the AQP family members in oyster species.

**Fig. 8:**
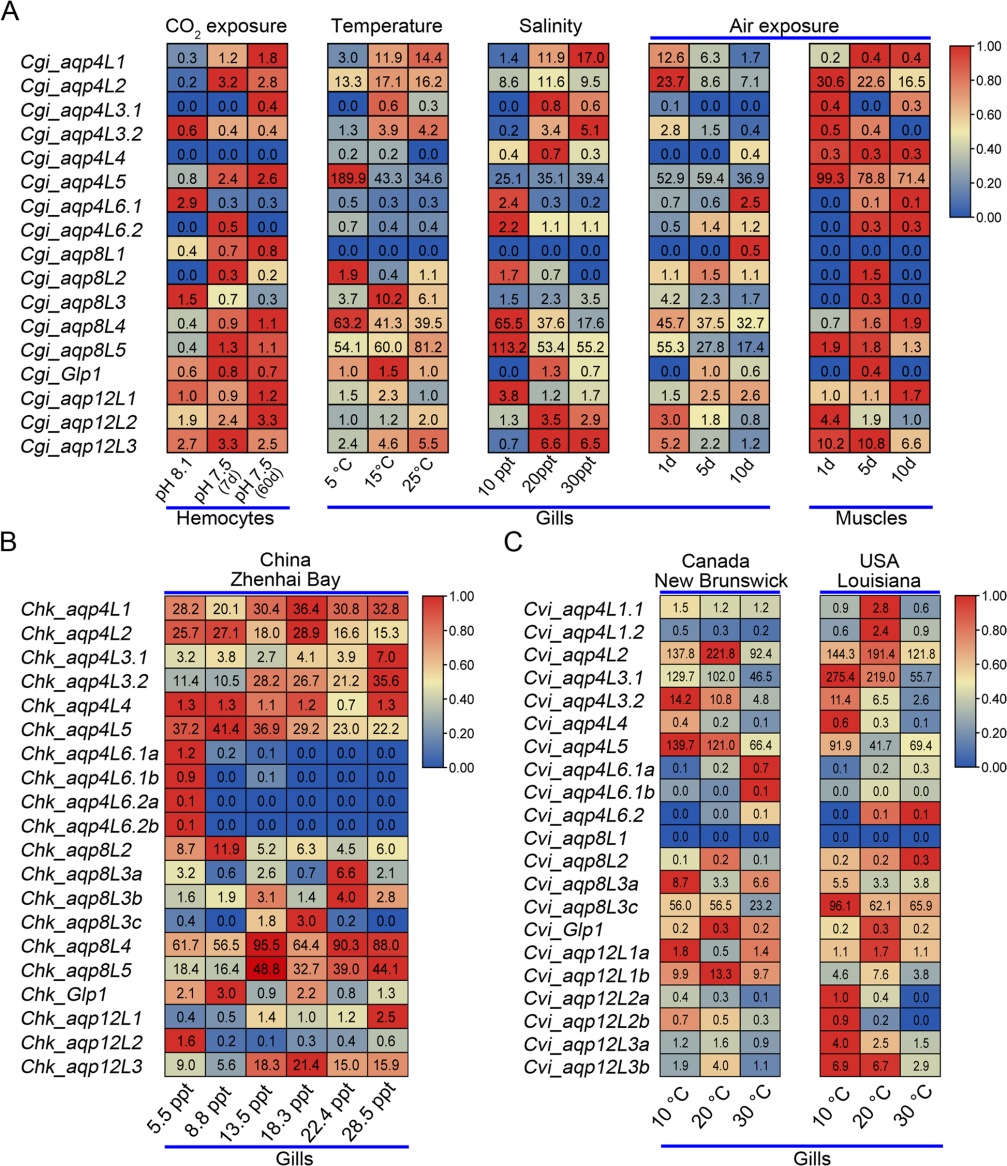
Diverse expression of the AQP family members in different oyster species. **A** Normalized expression levels of the AQP family members in Pacific oyster after the abiotic challenges including temperature, salinity and air exposure. **B** Normalized expression levels of the AQP family members in different Hong Kong oyster populations that distributed along the naturally formed salinity gradients. **C** Normalized expression levels of the AQP family members in separated eastern oyster populations that localized at the extremes of the geographic range along the Atlantic Ocean and acclimated at different temperatures

For molluscs that contain an open circulatory system, the organs directly bathed into the hemocoel to acquire oxygen and nutrients, and there is no distinction between blood and interstitial fluid. Moreover, the concentrations of the soluble substance in hemolymph were also extremely sensitive to the sudden change of surrounding environmental factors. It should be noted that the expression pattern of the complete set of AQP family members in hemolymph appeared at a relative lower level when compared with that in the other tissues or organs. Unexpectedly, most of them showed high sensitivity to the variation of environmental factors. For instance, transcriptome from the hemocytes was performed to analyze the impacts of short-term (7 days) and long-term (60 days) CO_2_ exposure (pH 7.50) on Pacific oyster. It is obvious that the expression of most AQP family members were significantly changed when faced with these challenges (Fig. 8A). Generally, the change of expression pattern for most AQP orthologs in oyster were highly conserved in response to the short- and long-term CO_2_ exposure. There are even some members (like Aqp4L1, -4L2 and -4L5) that were not expressed or expressed at a relative low level in the blank condition were significantly stimulated after the CO_2_ exposure treatment (Fig. 8A). In contrast, the expressions of some members (like Aqp4L6.1 and Aqp8L3) were significantly suppressed after the same treatment (Fig. 8A). However, whether the changes of AQP expression pattern are direct responses to these treatments or are responses to the metabolic disturbance that caused by these treatments still needs further investigation.

As an organ that serves as a tool for the filtering of water and gas exchange, gill is another organ with the highest exposure to the surrounding environments for the bivalve molluscs. In this context, gill also serves as the main interface between the organism and the environment. It should be noted that a majority of the AQP family members in gill showed a relative higher expression level when compared with that in the other organs in oyster (Additional file 6: Figure S6). In this context, the expression pattern of the AQP family members in gills in response to the environmental factors involving water temperature, salinity and air exposure were analyzed by utilizing the transcriptome data. Of the AQP orthologs in oyster gills, approximately half of them were differentially expressed under these abiotic stresses (Fig. 8A). It should be noted that the expressions of some members were specially changed in response to certain stresses. However, some members whose expression patterns were significantly changed were highly conserved in different treatment groups, especially between the groups that treated with salinity change and air exposure, in which the osmotic balance within the body’s fluids were disordered. Besides, the expression pattern of the tandem duplicated AQP orthologs were also highly diversified in response to different abiotic stresses. These data revealed the complexity and significant functional divergence of the abundant AQP family members in response to the dynamic environmental factors.

Apart from the samples that were collected from the same location and treated with different abiotic stresses that described above, transcriptome data for some geographically-distant oysters that native distributed to different environmental conditions were also collected to perform the different expression analysis. Comparison of the transcriptome data from six Hong Kong oyster populations that collected from distinct points in the Zhenhai Bay in Guangdong province, China, where the salinity gradients are naturally formed along the coast was conducted (Additional file 7: Figure S7A). It is clear that distinct expression profiles for some AQP genes were found among oyster populations that living across the naturally formed salinity gradients, especially between the populations that distributed in the extreme conditions (Fig. 8B). Moreover, the expression patterns of some members were also gradually changed combined with the salinity gradients. However, some others were irregular expressed, especially among the populations collected from the middle locations (P2 to P5). The most striking feature is that Aqp4L6 orthologs were just slightly expressed in the population that distributed under near freshwater condition. Similar profiles for these orthologs were also detected in the Pacific oyster that were treated with lower salinity (Fig. 8A). Additionally, the expression patterns of the tandem duplicated AQPs, especially for the members in the AQP8 subfamily, were also highly diversified among these distinct distributed Hong Kong oyster populations. These data demonstrate the significance of the duplication and differentiation of the AQP family members in response to the dynamic environmental factors.

Moreover, comparison of transcriptome for two geographically-distant eastern oyster populations that collected from either end of the species’ geographic range and occupy contrasting temperature and salinity habitats was conducted (Additional file 7: Figure S7B). Intriguingly, a highly diversified expression patterns for these AQP family members were obtained from these eastern oyster populations after the long-term maintains at the experimental salinity and temperature combination (Fig. 8C). It should be noted that the trend of expression profiles for some AQPs under different thermal regimes show no differences in physiological response to temperature between these two populations. Besides, the trend of expression patterns for some members even consistent with that for the orthologs in Pacific oysters that were treated with different temperatures (like Aqp4L5). Unexpectedly, some orthologs (like Aqp4L2, the duplicated -4L3.1 and duplicated Aqp8L3s) that were not expressed or just slightly expressed in the gills in Pacific oyster and Hong Kong oyster were highly expressed in eastern oysters. Moreover, these highly expressed orthologs were definitely sensitive to the variation of thermal regimes. Similarly, whether the changes of expression level for some AQP orthologs in oysters are direct responses to the environmental factors or are responses to the disorder of metabolism that caused by these treatments still needs further research.

## Discussion

The research herein represents a comprehensive set of genomic data that revealing a massive duplication and subsequent lineage-specific diversification of the AQP family in oyster species. As a selective channel that mediated the permeation of water molecules and some other nonionic solutes and even gasses, diversification of the AQP family plays critical roles in multiple physiology and pathophysiology processes in living organisms, especially for the aquatic species [9]. Although four groups that represent the separated subfamilies were distinguished within the AQP family, phylogenetic analyses have revealed that the diversified AQP subfamily members may share the same ancestor in prokaryotes [30]. Whole genome duplications, local gene duplications and even horizontal gene transfer events that occurred during the process of evolution are the basis for the diversification and functional differentiation of the AQP superfamily with adaptive selections in response to the environment changes in multicellular organisms [9, 10]. The unexpected expansion of the AQP family in oysters provides the feasibility to reveal the functional differentiation of the duplicated members in adaptation to the dynamic environmental factors.

WGD events were proposed to be the key roles to drive the phenotypic complexity, functional novelty and ecological adaptation during the process of evolution [31, 32]. However, only a few WGD events occurred in animals when compared with that in plants [33]. Previous research has revealed that two rounds of WGD events that occurred in the common ancestor of vertebrates have remarkably increased the total number of AQPs and shaped the adaptive radiation in vertebrates [15]. As the second largest phylum in all living animals on earth, molluscs inhabit extensive diversified environments including terrestrial, marine and freshwater habitats. However, only a few WGD events have occurred in molluscs [22]. It is clear that instead of WGD, tandem duplication has extensively increased the AQP members in oysters (Fig. 1). Unexpectedly, except for the contracted Glp subfamily that contained only one member, the other three subfamilies were all highly diversified in oyster species. Combined with the distribution and duplication pattern of the AQPs in gastropods, we could deduce that tandem duplication was the main method for the diversification of AQPs in molluscs.

Intriguingly, the low collinearity of the gene arrangement between gastropods and bivalves indicated that the diversification of the AQP family were independently evolved in molluscs. Moreover, synteny analysis revealed that several independent tandem duplication events and even inner chromosome inversions lead to the diversification of the AQP family in different oyster species (Fig. 3 and 4). Additionally, several orthologs with atypical Ar/R region, which was regarded as the basis for functional differentiation, have been detected in the AQP family in oysters (Fig. 5). Previous researches have revealed that some AQP orthologs are specific to molluscs only [23]. Besides, the molluscs typical AQPs could be distinguished into two specific groups and were named as malacoaquaporins (Maqp) and malacoglyceroporins (Mglp) independently. While considering the diversification of molluscs species, the occurrence and functional differentiation of these specific AQP orthologs still require further research.

The distinguishing feature of Maqp is the replacements of the conserved Phe and His in the first two sites in the Ar/R region [24]. It is noteworthy that a similar composition pattern of the Ar/R region was also detected in Aqp4L1 orthologs in oysters (Fig. 5). The conserved first two sites of the Ar/R region were all replaced by the amino acids with hydrophobic side chains. Generally, the AQPs that contain a similar composition pattern of the Ar/R region typically appeared in plants NIP subfamily that was specialized from classical AQP subfamily [34]. It should be noted that except for the classical AQP subfamily, the other three subfamilies were all absent in plants. In this context, the diversification of the classical AQPs in plants functioned as compensation for the absence of the other subfamilies [35]. It is well known that the imidazole ring in the side chain of His in the Ar/R region could construct a hydrogen bond with water molecules. Besides, the conserved Phe in the Ar/R region could also extremely enhance the hydrophily of the pore in this segment [36]. The amino acid replacements on these two sites in these atypical classical AQPs definitely changed the affinity of the channel inner surface to the water molecules [37]. Additionally, these replacements could also enlarge the diameter of the pore in the Ar/R region and accommodate the passage of large molecules. For instance, the NIPs in plants were reported to facilitate the permeation of glycerol, silicon and even boron other than water [38].

Except that, similar AQPs in the classical AQP subfamily also appeared in almost all living vertebrates except hagfishes and eutherian mammals and some invertebrates including molluscs [10, 39]. While unlike the absence of three AQP subfamilies in plants, all of the four AQP subfamilies were present in the species mentioned above. Similar atypical classical AQPs in teleost were reported to play osmoregulatory roles in adaptation to the seawater by facilitating the permeation properties elicited by aquaglyceroporins [39]. However, similar permeation properties were not detected in molluscs. For instance, previous research revealed that although these replacements enlarged the diameter of the AQP ortholog in Pacific abalone, it was still not capable of mediating the permeation of glycerol [40]. Besides, similar orthologs in terrestrial snail *Helix pomatia* could stimulate the permeation of hydrogen peroxide but is not permeable to glycerol [24]. It could be deduced from the relative position of the AQP family members on the chromosome that the atypical orthologs that are similar to Aqp4L1 were independently evolved in vertebrates and molluscs. In this context, the function of the atypical AQP orthologs among the species in different phylums were also highly diversified. It should be noted that the Aqp4L1 orthologs were diversely expressed in different oysters (Fig. 8), which may be attributed to the genetic effects. Interestingly, both of the duplicated Aqp4L1 orthologs in eastern oyster were slightly expressed in the gills of the two geographically-distant populations (Fig. 8C). However, the expression of the consistent orthologs in Hong Kong oysters were maintained at a higher level among six populations that distributed along the salinity gradients and no significant difference have been detected (Fig. 8B). Additionally, the consistent orthologs in Pacific oysters present a highly sensitive to various environmental factors. Unfortunately, the permeation properties and the physiological role of the atypical AQPs in oysters have not yet been explained. The physiological significance of this orthologs in oysters still needs further investigation.

The character of the Mglp orthologs that just appeared in gastropods was the replacement of the conserved His that occurred on the second site in the Ar/R region [23, 25]. However, the appearance of Mglp was not well supported in different studies [41]. In contrast to that in Aqp4L1 in oysters, the conserved Phe in the first site of the Ar/R region were retained in Mglps [24]. Remarkably, some AQPs with the similar composition pattern of the Ar/R region to that in Mglp also appeared in holometabolan insects to compensate for the absence of Glp subfamily in these species [26]. Previous research has revealed that the Mglps in Pacific abalone were derived from tandem duplication and subsequent functional differentiation [40]. However, similar phenomenon was not detected between the tandem duplicated AQP orthologs in oysters, although the Glp subfamily in these species were extremely contracted. The variable composition of the Ar/R region indicated the higher diversification of the AQP family in molluscs.

The tandem duplicated Aqp4L3 orthologs were another atypical AQPs that clustered into the classical AQP subfamily in oysters. However, it was clearly distinguished from the Maqp and Mglp in molluscs. Besides, some AQPs that contain the identical composition pattern of the amino acids in the Ar/R region have never been reported in the other species based on the MIPModDB database [42]. The most intriguing feature for the Aqp4L3 orthologs in oysters was the tandem duplication events that independently occurred after the speciation. It should be noted that the consistent orthologs in Pacific oysters were rarely expressed in various organs (Additional file 6: Figure S6). While the expression level of these two duplicated orthologs were sensitive affected by the environmental factors (Fig. 8A). Similar expression profiles also occurred in Hong Kong oysters (Fig. 8B). It is clear that the expression patterns of these two duplicated orthologs showed a consistent trend that conserved with range of naturally formed salinity gradients (Fig. 8B). Unexpectedly, one of these duplicated orthologs in eastern oysters was expressed at an extremely high level when compared with that in the other oyster species (Fig. 8C). Additionally, the consistent orthologs also show high sensitivity to the salinity change in the population that collected from Louisiana that characterized by water salinity seasonally varies from 1 to 28 [43]. These phenomena revealed that the Aqp4L3 orthologs in oysters may play critical roles in response to extreme osmotic stresses, although the permeation properties and physiological role of these orthologs in oysters has not yet been explained.

Noteworthily, similar composition patterns like “T–R” were also detected in some prokaryotic bacteria and plants. Nevertheless, the similar composition characteristic of the AQP family in bacteria and plants was the absence of some subfamilies in the genome [9]. In this context, the presence of some atypical classical AQP orthologs in these species were differentiated and considered to function as a compensation for the absence of some subfamilies [34, 44, 45]. While considering the presence of the four subfamilies in oysters, we could deduce that the Aqp4L3 orthologs did not function as compensation for the absence of the others. Besides, similar orthologs were also not detected in the other molluscs. Remarkably, most specialized AQP subfamilies in plants were thought to be derived from horizontal gene transfer from bacteria [46, 47]. These phenomena give us a clue that the presence of these atypical AQP orthologs in oysters may also come from bacteria by horizontal gene transfer. The appearance of the atypical AQPs in oysters made the integration of molluscs AQP family in evolutionary context more complicated.

Abundant duplication and subsequent diversification that occurred on the amino acids composition of the Ar/R region was the outstanding signature for the AQP8s in oyster species. Strikingly, except for the first site in the Ar/R region, the composition pattern of the other amino acids in the rest sites in Aqp8L1 was consistent with that in the duplicated Aqp4L3 orthologs in oysters (Fig. 5). However, similar AQP orthologs were exceedingly rare in the other molluscs and even pseudogenized in Hong Kong oysters. Besides, the retained orthologs in Pacific oysters and eastern oysters were just trace expressed in various conditions. These data revealed that the Aqp8L1 orthologs does not play any role in adaptation to the environmental stressors in oyster species. It should be noted that the altered amino acids in the Ar/R region between these two orthologs were all belonging to the group that the side chains were polar uncharged. This data suggested the character of the inner surface in this segment may not be changed between these orthologs despite the replacement of these amino acids. While combining the difference of the corresponding loops in length, the replacement in this site may lead to the variation of the inner surface in diameter between these two AQP orthologs (Figs. 6 and 7). Noteworthily, some composition patterns of the Ar/R region like “Q–R” were also widely reported in bacteria and plants [42]. These data suggested the frequent presence of the AQPs that are similar to the members that typically appeared in plants may be related to the filter-feeding habits of oysters.

Another feature of the abundant AQP8s in oysters was the absence of the two duplicated orthologs (Aqp8L4 and -8L5) in eastern oyster. Blast analysis revealed these two orthologs were also not detected in Sydney rock oyster (data not shown). These data revealed that the arise of these two duplicated AQP orthologs was independently occurred earlier than the speciation of the recent common ancestor of Pacific oyster and Hong Kong oyster in the evolution process. The uncertainty of the presence of these two duplicated AQP8 orthologs in different oysters suggested that it may be closely related to the variety of habitats. It is obvious that the two duplicated orthologs that retained in Pacific oysters and Hong Kong oysters were highly expressed when compared with the other members. Besides, the expression pattern of these two members also showed extreme sensitivities to the fluctuation of environmental factors. Considering the broad range of environmental conditions that eastern oysters inhabited, the absence of two duplicated AQP8s in this species may be compensated by the others. Unexpectedly, one of the independently duplicated Aqp8L3 orthologs in eastern oysters were expressed at a relative higher level when compared with that in the other oysters. However, the composition pattern of the amino acids in the Ar/R region in the Aqp8L3 orthologs were distinctly from that in the Aqp8L4 and -8L5 orthologs. The prominent characteristic of the Ar/R region in Aqp8L4 and -8L5 orthologs is the replacements of the conserved Arg that localized on the last site. It is notable that AQPs in which the conserved Arg in the fourth site of the Ar/R region was replaced by the other amino acids rarely appeared in the other three subfamilies except for that in unorthodox AQP subfamily [37]. As the side chain of Arg was positively charged in the normal range of the physiological pH environment, the conserved fourth site in the Ar/R region is the basis for the channel to function as a cation filter [20]. In this condition, the replacement of the amino acid in this site will deeply alter the function of the channel. Moreover, no alteration has been detected in the composition of the Ar/R region between these two orthologs after the tandem duplication events in oysters. It is clear that there are some other determination sites for functional differentiation in these two orthologs based on the difference of the channel inner surface (Fig. 7). Additionally, the difference of the last exon segment in length between these two members also play important roles in the selective permeation process. While nothing is known about their transport properties in oysters.

## Conclusions

In summary, massive expanded AQP families have been detected in oyster species. Phylogenetic analysis revealed that the expanded AQP family was extensively diversified and all of the four AQP subfamilies were presented in oysters. Chromosome distribution analysis indicated that tandem duplication and subsequent functional differentiation have created the remarkable diversification of the AQP family in oysters. Synteny analysis suggested that large scale inner chromosome inversion and independent tandem duplication events also lead to the variation of the AQP orthologs in different oyster species. Moreover, pore pattern analysis of the inner surface revealed that the highly variable of the amino acids in the Ar/R region are the basis for the functional differentiation between the tandem duplicated AQPs in oysters. Comparison that conducted on the transcriptome data revealed the functional complexity of expanded AQP orthologs in oyster species in response to the variety of abiotic environmental factors. Briefly, the present study revealed a comprehensive understanding of the diversification that occurred on the AQP family in oyster species. Additionally, it also provides essential genomic resources for further physiological and evolutionary study of the AQP family in molluscs.

## Methods

### Identification of the AQPs in oysters

Owing to the wide distribution of oysters on the earth, three species including *C. gigas*, *C. hongkongensis* and *C. virginica* were selected to perform the related analysis in this study. Available genome assemblies and the corresponding annotation files for these species were acquired from the NCBI public database (Additional file 8: Table S1). Based on the conservation of the NPA motif in the AQP sequences, the complete set of the AQP family members in these oysters were de novo identified using Hmmer (v3.3.2) [48] and Tblastn (v2.7.1) programs [49] with the default parameters. The complete set of the AQP family in Pacific abalone was regarded as queries to search against these three oyster genome sequences. Meanwhile, the transcriptomes for the adult tissues were collected from RNA-seq data for different oysters (Additional file 8: Table S1) and then de novo assembled by utilizing the Trinity (v2.12) software [50]. The identified AQP sequences in different oysters were revised based on the de novo assembled transcripts independently. To make the subsequent analysis and description more convenient, the AQP genes in Pacific oyster were named based on the chromosome location and the subfamily that they clustered. While the AQP genes in Hong Kong oyster and eastern oyster were also renamed based on the corresponding members in Pacific oyster.

## Bioinformatic analysis of the AQPs in oysters

To define the identified AQPs in oysters, phylogenetic analysis was performed with reference AQP protein sequences from the well characterized model species. Besides, the complete set of the AQP family in another well characterized mollusc, Pacific abalone, was also included to conduct this analysis. The complete set of the AQP genes in different species were initially aligned by utilizing the ClustalX (v2.1) program with the default parameters [51]. The phylogenetic dendrograms were constructed using the Mega (v7.0.26) program based on the alignment of the protein sequences with both Maximum-likelihood (ML) and Neighbor-joining (NJ) methods [52]. A total of 1000 bootstrap replicates were computed for different calculation. The nomenclature and annotation of the identified AQPs in oysters were performed based on the phylogenetic analysis. Besides, the alignment of the AQPs in oysters was visualized in Dnaman9 software (Lynnon Biosoft, CA, USA). The composition of the amino acids in the NPA motif and the Ar/R region in different AQP orthologs in oysters were summarized based on the multiple alignment database. To detected the duplication of the AQP family in oysters, the chromosome distribution of the family members was analyzed using Tbtools (v1.09) software based on the annotation information [53]. To detect the differentiation between the duplicated AQP orthologs, the exon/intron structures of the AQPs in Pacific oyster were performed by utilizing the Gene Structure Display Server (GSDS 2.0) online service [54] depending on the annotation file.

## Synteny analysis of the AQP family in different oysters

To explore the duplication and deletion of the AQP orthologs in these three different oysters, the synteny relationship between each of these two oysters were performed using the JCVI (v1.1.15) program based on the annotation files and the corresponding CDS sets for each oyster [55]. The collinearity relationship between the gene pairs were visualized using the Advanced circos program in Tbtools. Additionally, the micro-synteny relationship around the distribution of each AQP orthologs was visualized using the find gene block evolutionary path by gene pairs program in Tbtools. Moreover, the pseudogenes in each oyster were detected using the local Blastn program to reveal the deletion process. The corresponding AQP orthologs were used as queries to blast against the DNA sequences around the pseudogenes. The blast results were visualized using Dnaman9 software.

## Homology modeling and pore pattern analysis

To detect the influence of the variable amino acids in the Ar/R region on the inner surface pattern of the channel, the three-dimensional structures of the AQP orthologs in Pacific oyster were predicted using the Swiss-Model online server with the default parameters [56]. The structure of classical AQPs were predicted based on the template of human AQP-4 (PDB ID: 3gd8). The structure of members in the AQP8 subfamily were constructed based on the crystal structure of AtTIP2;1, an ammonia permeable AQP in *Arabidopsis thaliana* (PDB ID: 5i32). The location of the amino acids in the Ar/R region in the constructed structure were displayed using the PyMol (v2.2.2) molecular visualization system [57]. The inner-surface of the AQP orthologs in oysters were calculated by utilizing the Hole2 (v2.2.005) program based on the predicted structures [58]. The produced channel profiles of the AQP orthologs were visualized using the VMD (v1.9.2) software [59].

## Expression calculation of the AQP family members

The transcriptome data used in present study were summarized in Additional file 8 (Table S1). For Pacific oysters, transcriptome data from different developmental stages and different organs or tissues were downloaded from the NCBI database under the BioProject number PRJNA146329 [3]. Besides, the transcriptome data from the organs that treated under various environmental stressors (involving exposure to temperature, salinity and air) for this species were also collected from the NCBI database under the BioProject number PRJNA146329 [3]. For Hong Kong oysters, which exhibits remarkable eurysalinity traits, have acquired a powerful capacity for adapting to the inherently dynamic estuarial habitats, and thrive within salinity gradients range from 5 to 30 ppt. In this context, transcriptome data from six populations that distributed along the naturally formed salinity gradients in Zhenhai Bay in southern China (Additional file 7: Figure S7A) were obtained from the NCBI database under the BioProject number PRJNA488328 [60]. For eastern oysters that geographic distributed from Gulf of St. Lawrence to the Gulf of Mexico and Caribbean Sea and inhabits an especially broad range of conditions including temperature and salinity, the separately distributed populations are each optimized to their individual conditions [61]. Transcriptome data from two eastern oyster populations that collected close to the extremes of the geographic range (Additional file 7: Figure S7B) and acclimated with different temperature were downloaded from the NCBI database under the BioProject number PRJNA719567 [61]. Clean reads were obtained by filtering the raw data with the Trimmomatic v0.39 software [62]. And then, the clean reads were first mapped to the corresponding genomes using HISAT2 v2.2.1 software [63]. The alignments were then passed to the StringTie v2.1.4 software, which assembled and quantified the transcripts in each sample [64]. Gene expression levels of the AQP family members in different oysters were extracted from the profiles that were normalized to Fragments per Kilobase per Million (FPKM). Heatmaps for these expression profiles that normalized by considering the maximum expression levels for each gene in the same tissue were generated using TBtools v1.09 [53].

## Supplementary Information

**Additionalfile 1: FigureS1. **Identities between the AQP protein sequences in three oyster species. (A)Identity between the complete set of the AQP family members in oysters. (B)Partial enlarged view of identities between the duplicated Aqp4L3 orthologs inoysters. (C) Partial enlarged view of identities between the duplicated Aqp4L6orthologs in oysters. (D) Partial enlarged view of identities between theduplicated AQP8 orthologs in oysters. (E) Partial enlarged view of identitiesbetween the duplicated orthologs in the S-AQP subfamily in oysters.

**Additionalfile 2: Figure S2. **Synteny analysis between different oyster species. (A) Chromosome-level syntenybetween each of these oysters. (B) Correlation of the AQP family members indifferent oyster species.

**Additionalfile 3: Figure S3. **Pseudogenization of the duplicated AQP orthologs in oysters. (A) Blast analysisof the pseudogenization that occurred after the duplication of Aqp4L1 inPacific oyster and Hong Kong oyster. (B) Blast analysis of the pseudogenizationthat occurred after the duplication of Aqp4L5 ortholog in Pacific oyster. (C)Blast analysis of the pseudogenization that occurred after the duplication ofAqp4L6 ortholog in Hong Kong oyster.(DOCX 5911 kb)

**Additionalfile 4: Figure S4. **Pseudogenization of the duplicated AQPs in oysters. (A) Blast analysis of thepseudogenization that occurred after the duplication of Glp1 in Hong Kongoyster. (B) Blast analysis of the pseudogenization that occurred after theduplication of Aqp8L1 in Hong Kong oyster. (C) Insertion that occurred in thethird exon in the ψChk_aqp8L1c in Hong Kong oyster. (D) Genome widedistribution of the segments that similar to the inserted region in the thirdexon in the ψChk_8.1c in Hong Kong oyster.

**Additionalfile 5: Figure S5. **Multiple alignments of the protein sequences in the AQP8 subfamily in oysters.The alignments were separated by the exons. Except the conserved second exonregion, the others were highly varied between the tandem duplicated orthologs.

**Additionalfile 6: Figure S6. **Heatmaps of expression for the AQP family members at different developmentalstages and in different organs in Pacific oyster.

**Additionalfile 7: Figure S7. **Geographocal distribution of the oysters that collected for transcriptomeanalysis. (A) Sampling locations of the Hong Kong oyster populations acrosssalinity gradients in Zhenhai Bay. (B) Sampling location of the eastern oysterpopulations at the extremes of the geographic range along the Atlantic Ocean.

**Additionalfile 8: Table S1. **Summary of the data that collected from NCBI in this study.

## Data Availability

All data generated or analysed during this study are included in this published article and its supplementary information files.
