# Supplementary material for: Diversification of the aquaporin family in geographical isolated oyster species promote the adaptability to dynamic environments
Source: BMC Genomics. 2022 Mar 16;23:211. doi: 10.1186/s12864-022-08445-4 (PMC8925068; doi:10.1186/s12864-022-08445-4)

**Additional files**

**Additional file 1: Figure S1** Identities between the AQP protein sequences in three oyster species. (A) Identity between the complete set of the AQP family members in oysters. (B) Partial enlarged view of identities between the duplicated Aqp4L3 orthologs in oysters. (C) Partial enlarged view of identities between the duplicated Aqp4L6 orthologs in oysters. (D) Partial enlarged view of identities between the duplicated AQP8 orthologs in oysters. (E) Partial enlarged view of identities between the duplicated orthologs in the S-AQP subfamily in oysters.


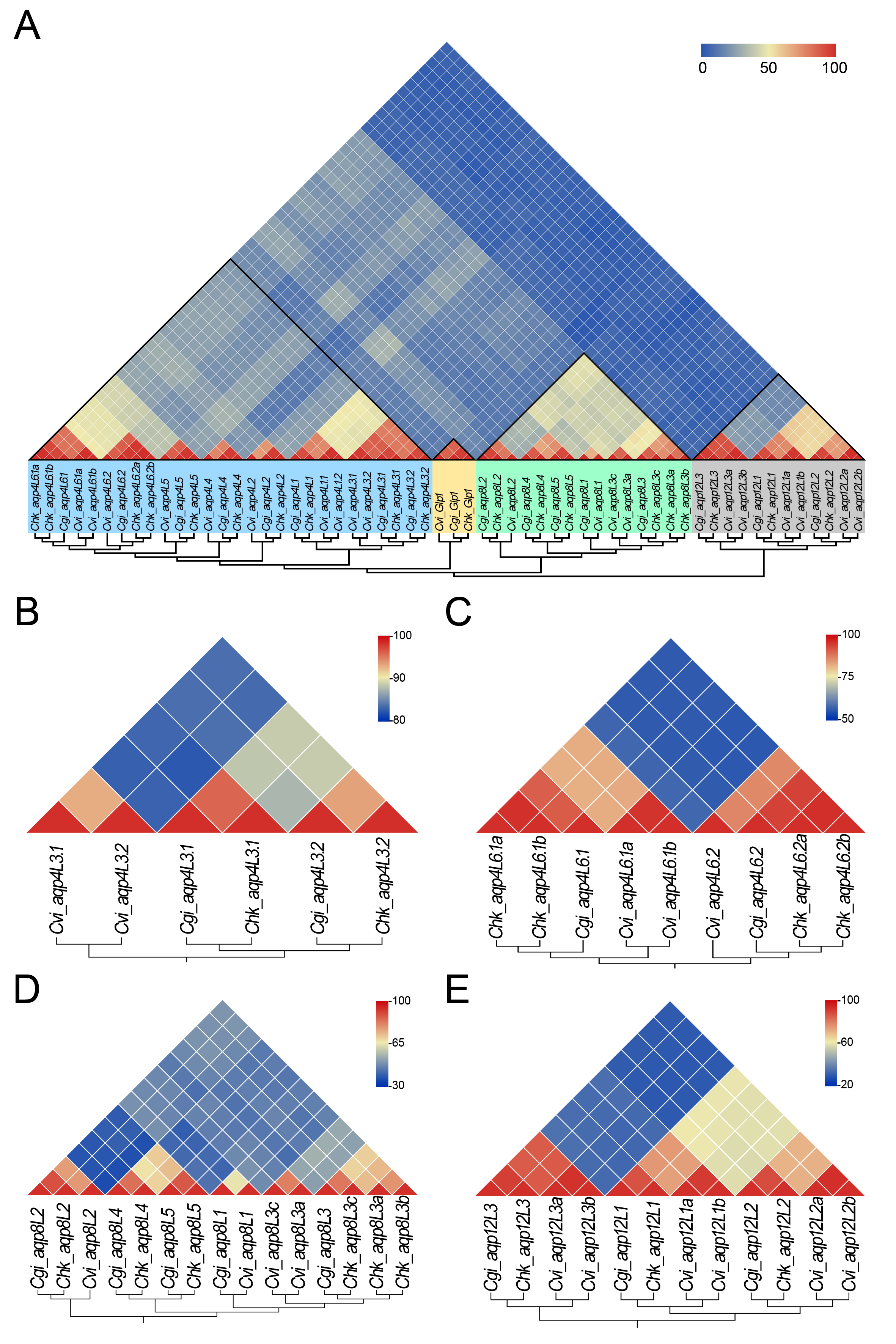

Supplement: Supplementary file 1 — Additionalfile 1: FigureS1. Identities between the AQP protein sequences in three oyster species. (A)Identity between the complete set of the AQP family members in oysters. (B)Partial enlarged view of identities between the duplicated Aqp4L3 orthologs inoysters. (C) Partial enlarged view of identities between the duplicated Aqp4L6orthologs in oysters. (D) Partial enlarged view of identities between theduplicated AQP8 orthologs in oysters. (E) Partial enlarged view of identitiesbetween the duplicated orthologs in the S-AQP subfamily in oysters. [file 12864_2022_8445_MOESM1_ESM.docx]
