# Supplementary material for: Diversification of the aquaporin family in geographical isolated oyster species promote the adaptability to dynamic environments
Source: BMC Genomics. 2022 Mar 16;23:211. doi: 10.1186/s12864-022-08445-4 (PMC8925068; doi:10.1186/s12864-022-08445-4)

**Additional file 2: Figure S2** Synteny analysis between different oyster species. (A) Chromosome-level synteny between each of these oysters. (B) Correlation of the AQP family members in different oyster species.


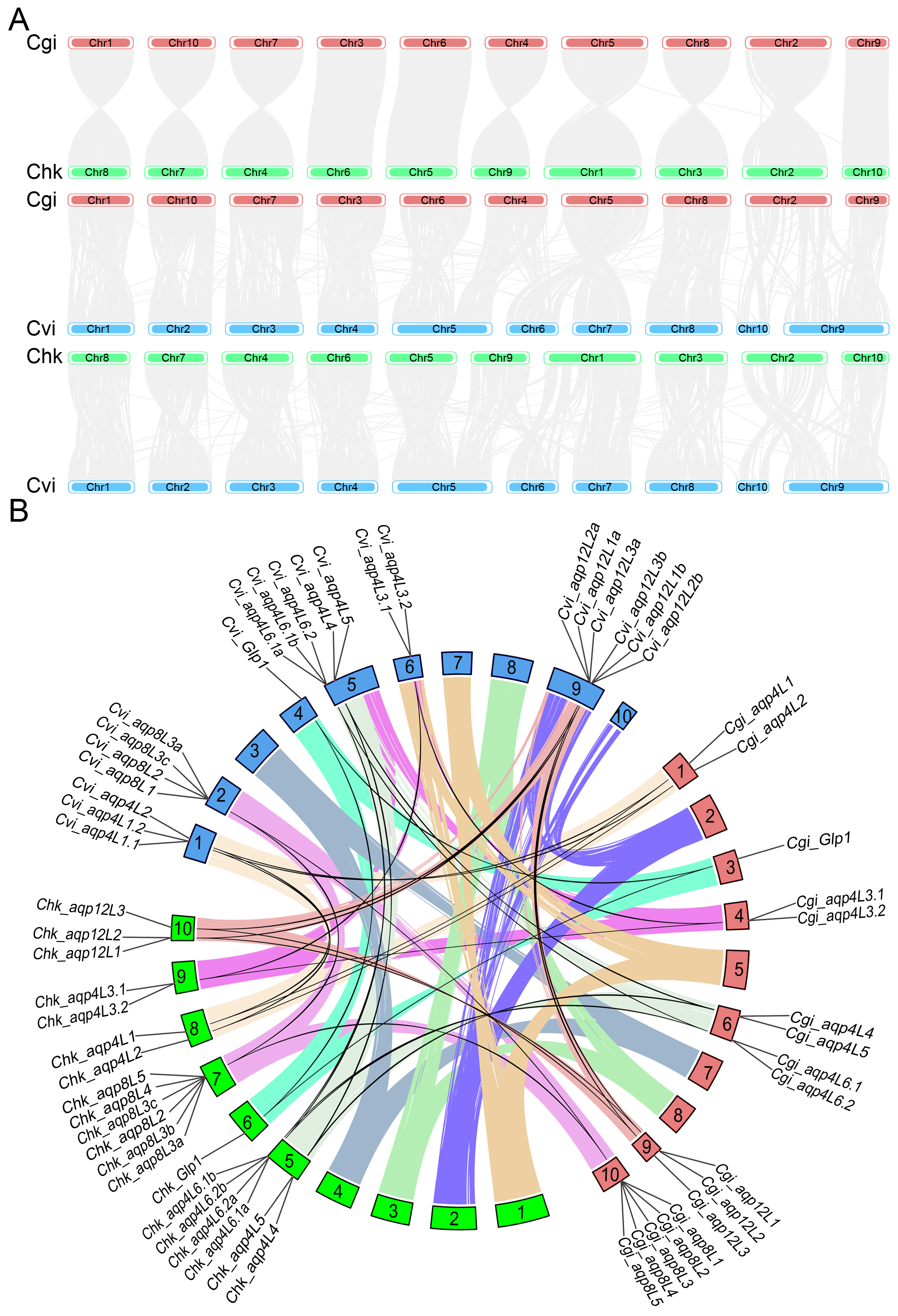

Supplement: Supplementary file 2 — Additionalfile 2: Figure S2. Synteny analysis between different oyster species. (A) Chromosome-level syntenybetween each of these oysters. (B) Correlation of the AQP family members indifferent oyster species. [file 12864_2022_8445_MOESM2_ESM.docx]
