# Supplementary material for: Diversification of the aquaporin family in geographical isolated oyster species promote the adaptability to dynamic environments
Source: BMC Genomics. 2022 Mar 16;23:211. doi: 10.1186/s12864-022-08445-4 (PMC8925068; doi:10.1186/s12864-022-08445-4)

**Additional file 3: Figure S3** Pseudogenization of the duplicated AQP orthologs in oysters. (A) Blast analysis of the pseudogenization that occurred after the duplication of Aqp4L1 in Pacific oyster and Hong Kong oyster. (B) Blast analysis of the pseudogenization that occurred after the duplication of Aqp4L5 ortholog in Pacific oyster. (C) Blast analysis of the pseudogenization that occurred after the duplication of Aqp4L6 ortholog in Hong Kong oyster.


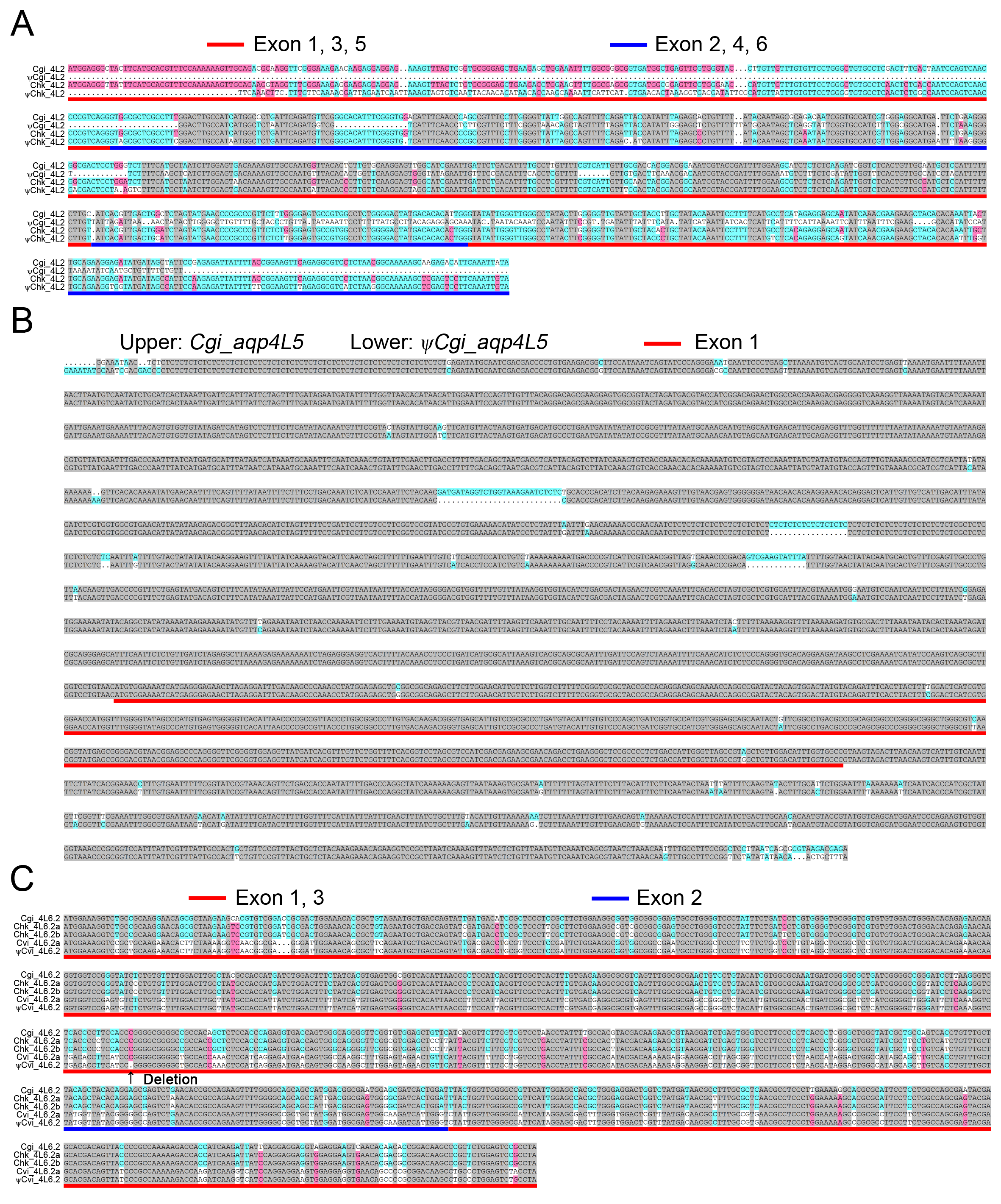

Supplement: Supplementary file 3 — Additionalfile 3: Figure S3. Pseudogenization of the duplicated AQP orthologs in oysters. (A) Blast analysisof the pseudogenization that occurred after the duplication of Aqp4L1 inPacific oyster and Hong Kong oyster. (B) Blast analysis of the pseudogenizationthat occurred after the duplication of Aqp4L5 ortholog in Pacific oyster. (C)Blast analysis of the pseudogenization that occurred after the duplication ofAqp4L6 ortholog in Hong Kong oyster.(DOCX 5911 kb) [file 12864_2022_8445_MOESM3_ESM.docx]
