# Supplementary material for: Diversification of the aquaporin family in geographical isolated oyster species promote the adaptability to dynamic environments
Source: BMC Genomics. 2022 Mar 16;23:211. doi: 10.1186/s12864-022-08445-4 (PMC8925068; doi:10.1186/s12864-022-08445-4)

**Additional file 4: Figure S4** Pseudogenization of the duplicated AQPs in oysters. (A) Blast analysis of the pseudogenization that occurred after the duplication of Glp1 in Hong Kong oyster. (B) Blast analysis of the pseudogenization that occurred after the duplication of Aqp8L1 in Hong Kong oyster. (C) Insertion that occurred in the third exon in the ψChk_aqp8L1c in Hong Kong oyster. (D) Genome wide distribution of the segments that similar to the inserted region in the third exon in the ψChk_8.1c in Hong Kong oyster.


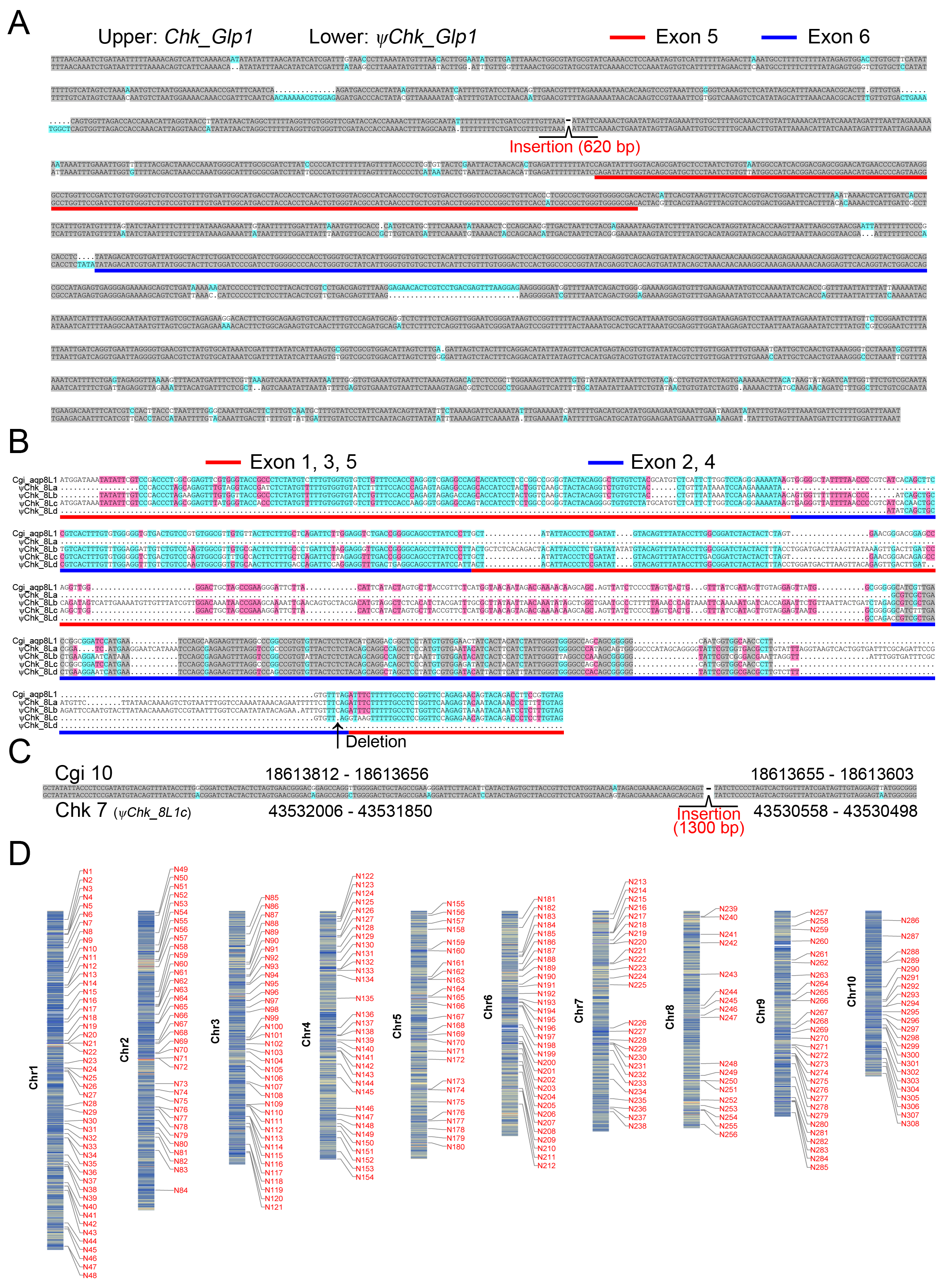

Supplement: Supplementary file 4 — Additionalfile 4: Figure S4. Pseudogenization of the duplicated AQPs in oysters. (A) Blast analysis of thepseudogenization that occurred after the duplication of Glp1 in Hong Kongoyster. (B) Blast analysis of the pseudogenization that occurred after theduplication of Aqp8L1 in Hong Kong oyster. (C) Insertion that occurred in thethird exon in the ψChk_aqp8L1c in Hong Kong oyster. (D) Genome widedistribution of the segments that similar to the inserted region in the thirdexon in the ψChk_8.1c in Hong Kong oyster. [file 12864_2022_8445_MOESM4_ESM.docx]
