# Supplementary material for: Diversification of the aquaporin family in geographical isolated oyster species promote the adaptability to dynamic environments
Source: BMC Genomics. 2022 Mar 16;23:211. doi: 10.1186/s12864-022-08445-4 (PMC8925068; doi:10.1186/s12864-022-08445-4)

**Additional file 5: Figure S5** Multiple alignments of the protein sequences in the AQP8 subfamily in oysters. The alignments were separated by the exons. Except the conserved second exon region, the others were highly varied between the tandem duplicated orthologs.


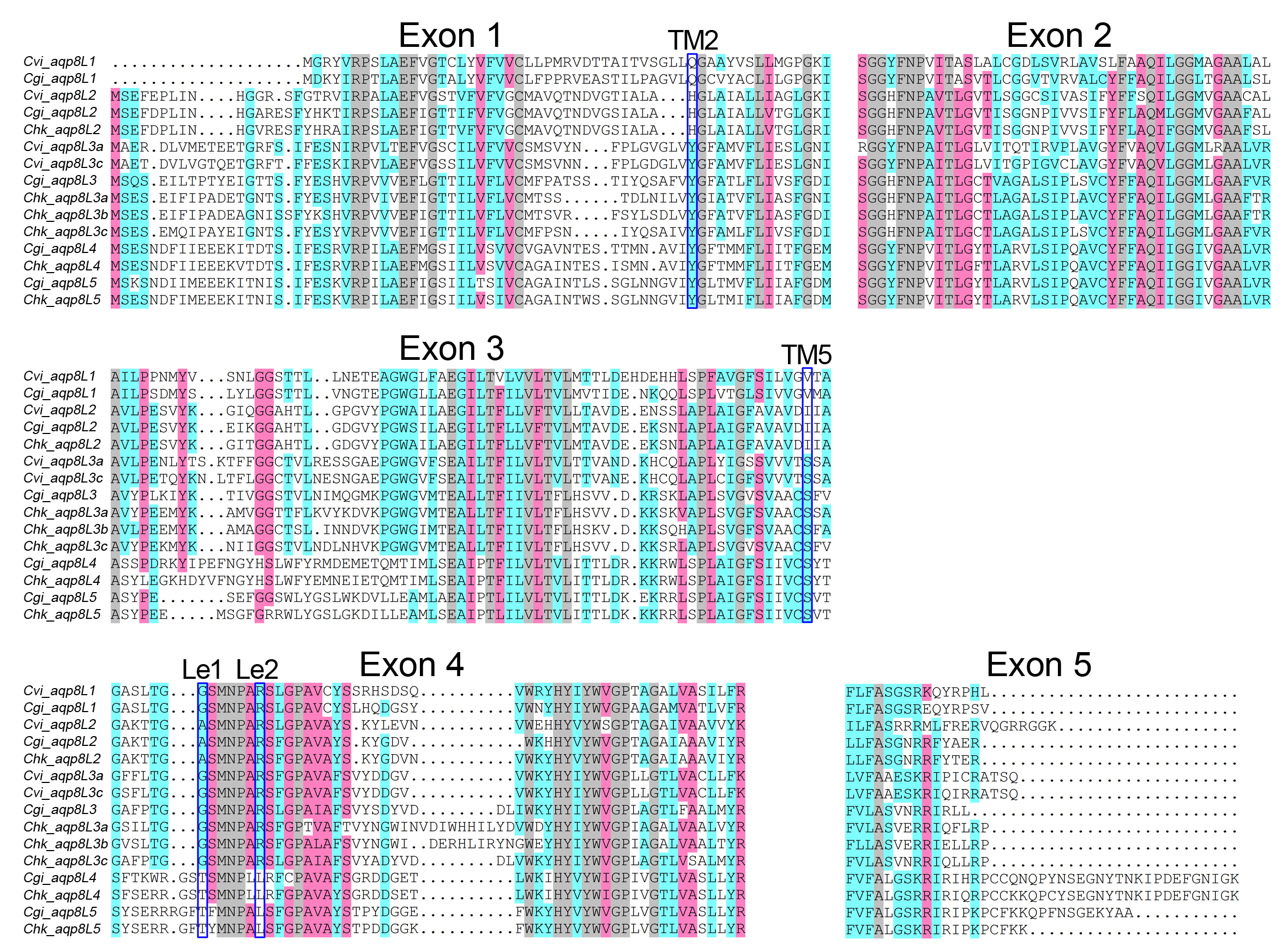

Supplement: Supplementary file 5 — Additionalfile 5: Figure S5. Multiple alignments of the protein sequences in the AQP8 subfamily in oysters.The alignments were separated by the exons. Except the conserved second exonregion, the others were highly varied between the tandem duplicated orthologs. [file 12864_2022_8445_MOESM5_ESM.docx]
