# Supplementary material for: Diversification of the aquaporin family in geographical isolated oyster species promote the adaptability to dynamic environments
Source: BMC Genomics. 2022 Mar 16;23:211. doi: 10.1186/s12864-022-08445-4 (PMC8925068; doi:10.1186/s12864-022-08445-4)

**Additional file 6: Figure S6** Heatmaps of expression for the AQP family members at different developmental stages and in different organs in Pacific oyster.


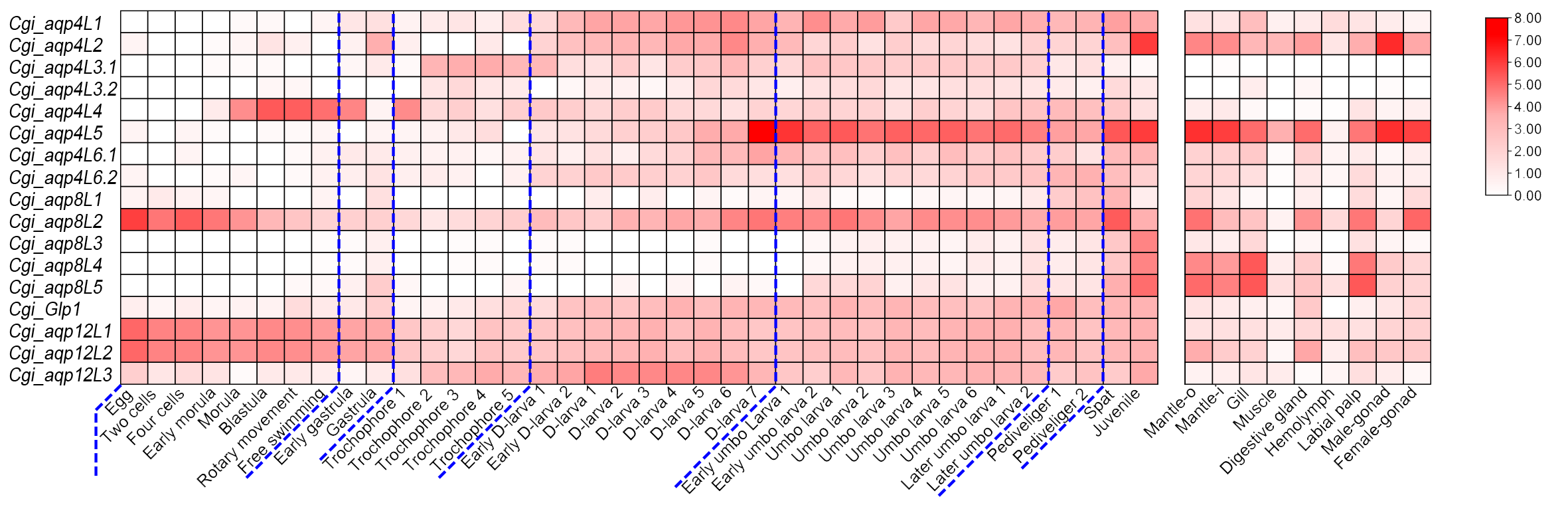

Supplement: Supplementary file 6 — Additionalfile 6: Figure S6. Heatmaps of expression for the AQP family members at different developmentalstages and in different organs in Pacific oyster. [file 12864_2022_8445_MOESM6_ESM.docx]
