# Supplementary material for: Diversification of the aquaporin family in geographical isolated oyster species promote the adaptability to dynamic environments
Source: BMC Genomics. 2022 Mar 16;23:211. doi: 10.1186/s12864-022-08445-4 (PMC8925068; doi:10.1186/s12864-022-08445-4)

**Additional file 7: Figure S7** Geographocal distribution of the oysters that collected for transcriptome analysis. (A) Sampling locations of the Hong Kong oyster populations across salinity gradients in Zhenhai Bay. (B) Sampling location of the eastern oyster populations at the extremes of the geographic range along the Atlantic Ocean.


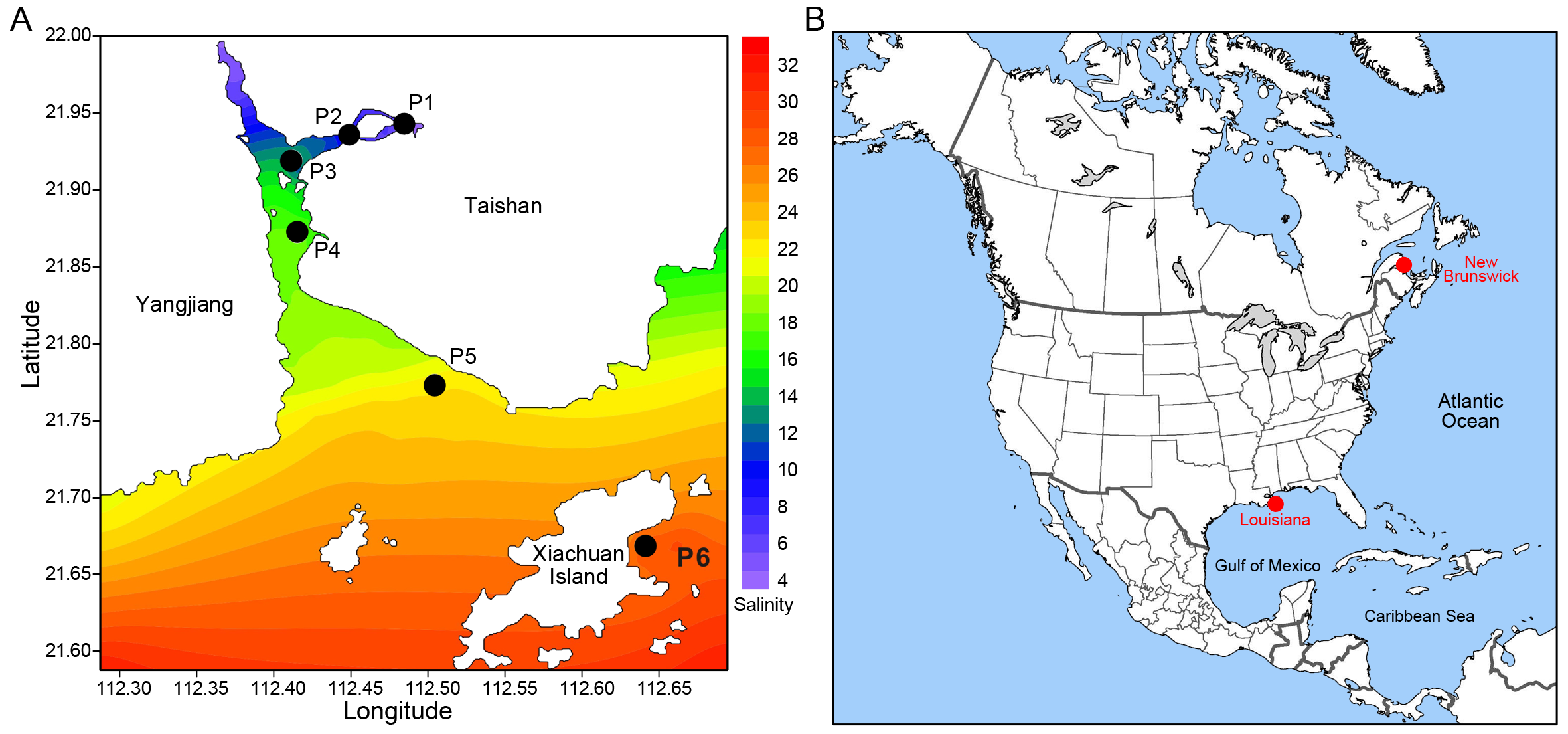

Supplement: Supplementary file 7 — Additionalfile 7: Figure S7. Geographocal distribution of the oysters that collected for transcriptomeanalysis. (A) Sampling locations of the Hong Kong oyster populations acrosssalinity gradients in Zhenhai Bay. (B) Sampling location of the eastern oysterpopulations at the extremes of the geographic range along the Atlantic Ocean. [file 12864_2022_8445_MOESM7_ESM.docx]
