# Supplementary material for: Diversification of the aquaporin family in geographical isolated oyster species promote the adaptability to dynamic environments
Source: BMC Genomics. 2022 Mar 16;23:211. doi: 10.1186/s12864-022-08445-4 (PMC8925068; doi:10.1186/s12864-022-08445-4)
Supplement: Supplementary file 8 — Additionalfile 8: Table S1. Summary of the data that collected from NCBI in this study. [file 12864_2022_8445_MOESM8_ESM.docx]

**Additional file 8: Table S1** Summary of the data that collected from NCBI in this study.

| **Organism** | **Library Source** | **Accession number** | **Description** |
| --- | --- | --- | --- |
| *Crassostrea gigas* | Genomic | GCF_902806645.1 | Genome assembly |
| *Crassostrea hongkongensis* | Genomic | GCA_016163765.1 | Genome assembly |
| *Crassostrea* *virginica* | Genomic | GCA_002022765.4 | Genome assembly |
| *Crassostrea gigas* | Transcriptomic | SRR526975 | Mixture of the adult tissues |
| *Crassostrea hongkongensis* | Transcriptomic | SRR6201765 | Mixture of the adult tissues |
| *Crassostrea virginica* | Transcriptomic | SRR10982739 | Larval pool whole body |
| *Crassostrea gigas* | Transcriptomic | SRR334212 | Outer edge of mantle |
| *Crassostrea gigas* | Transcriptomic | SRR334213 | Digestive gland |
| *Crassostrea gigas* | Transcriptomic | SRR334214 | Female gonad |
| *Crassostrea gigas* | Transcriptomic | SRR334215 | Gill |
| *Crassostrea gigas* | Transcriptomic | SRR334216 | Inner part of mantle |
| *Crassostrea gigas* | Transcriptomic | SRR334217 | Adductor muscle |
| *Crassostrea gigas* | Transcriptomic | SRR334218 | Hemolymph |
| *Crassostrea gigas* | Transcriptomic | SRR334219 | Labial palp |
| *Crassostrea gigas* | Transcriptomic | SRR334220 | Male gonad |
| *Crassostrea gigas* | Transcriptomic | SRR334222 | Egg |
| *Crassostrea gigas* | Transcriptomic | SRR334223 | Two cells |
| *Crassostrea gigas* | Transcriptomic | SRR334224 | Four cells |
| *Crassostrea gigas* | Transcriptomic | SRR334225 | Early morula stage |
| *Crassostrea gigas* | Transcriptomic | SRR334226 | Morula stage |
| *Crassostrea gigas* | Transcriptomic | SRR334227 | Blastula stage |
| *Crassostrea gigas* | Transcriptomic | SRR334228 | Rotary movement |
| *Crassostrea gigas* | Transcriptomic | SRR334229 | Free swimming |
| *Crassostrea gigas* | Transcriptomic | SRR334230 | Early gastrula stage |
| *Crassostrea gigas* | Transcriptomic | SRR334231 | Gastrula stage |
| *Crassostrea gigas* | Transcriptomic | SRR334232 | Trochophore1 |
| *Crassostrea gigas* | Transcriptomic | SRR334233 | Trochophore2 |
| *Crassostrea gigas* | Transcriptomic | SRR334234 | Trochophore3 |
| *Crassostrea gigas* | Transcriptomic | SRR334235 | Trochophore4 |
| *Crassostrea gigas* | Transcriptomic | SRR334236 | Trochophore5 |
| *Crassostrea gigas* | Transcriptomic | SRR334237 | Early Dshaped larva1 |
| *Crassostrea gigas* | Transcriptomic | SRR334238 | Early Dshaped larva2 |
| *Crassostrea gigas* | Transcriptomic | SRR334239 | Dshaped larva1 |
| *Crassostrea gigas* | Transcriptomic | SRR334240 | Dshaped larva2 |
| *Crassostrea gigas* | Transcriptomic | SRR334241 | Dshaped larva3 |
| *Crassostrea gigas* | Transcriptomic | SRR334242 | Dshaped larva4 |
| *Crassostrea gigas* | Transcriptomic | SRR334243 | Dshaped larva5 |
| *Crassostrea gigas* | Transcriptomic | SRR334244 | Dshaped larva6 |
| *Crassostrea gigas* | Transcriptomic | SRR334245 | Dshaped larva7 |
| *Crassostrea gigas* | Transcriptomic | SRR334246 | Early umbo larva1 |
| *Crassostrea gigas* | Transcriptomic | SRR334247 | Early umbo larva2 |
| *Crassostrea gigas* | Transcriptomic | SRR334248 | Umbo larva1 |
| *Crassostrea gigas* | Transcriptomic | SRR334249 | Umbo larva2 |
| *Crassostrea gigas* | Transcriptomic | SRR334250 | Umbo larva3 |
| *Crassostrea gigas* | Transcriptomic | SRR334251 | Umbo larva4 |
| *Crassostrea gigas* | Transcriptomic | SRR334252 | Umbo larva5 |
| *Crassostrea gigas* | Transcriptomic | SRR334253 | Umbo larva6 |
| *Crassostrea gigas* | Transcriptomic | SRR334254 | later umbo larva1 |
| *Crassostrea gigas* | Transcriptomic | SRR334255 | later umbo larva2 |
| *Crassostrea gigas* | Transcriptomic | SRR334256 | Pediveliger1 |
| *Crassostrea gigas* | Transcriptomic | SRR334257 | Pediveliger2 |
| *Crassostrea gigas* | Transcriptomic | SRR334258 | Spat |
| *Crassostrea gigas* | Transcriptomic | SRR334259 | Juvenile |
| *Crassostrea gigas* | Transcriptomic | SRR334262 | Gills, sampled at time 7d, 5 ºC |
| *Crassostrea gigas* | Transcriptomic | SRR334264 | Gills, sampled at time 7d, 15 ºC |
| *Crassostrea gigas* | Transcriptomic | SRR334266 | Gills, sampled at time 7d, 25 ºC |
| *Crassostrea gigas* | Transcriptomic | SRR334270 | Gills, sampled at time 12h, salinity 10 ppt |
| *Crassostrea gigas* | Transcriptomic | SRR334272 | Gills, sampled at time 12h, salinity 20 ppt |
| *Crassostrea gigas* | Transcriptomic | SRR334274 | Gills, sampled at time 12h, salinity 30 ppt |
| *Crassostrea gigas* | Transcriptomic | SRR334276 | Gills, sampled at time 1d, exposure to air |
| *Crassostrea gigas* | Transcriptomic | SRR334278 | Gills, sampled at time 5d, exposure to air |
| *Crassostrea gigas* | Transcriptomic | SRR334281 | Gills, sampled at time 10d, exposure to air |
| *Crassostrea gigas* | Transcriptomic | SRR334284 | Muscles, sampled at time 1d, exposure to air |
| *Crassostrea gigas* | Transcriptomic | SRR334286 | Muscles, sampled at time 5d, exposure to air |
| *Crassostrea gigas* | Transcriptomic | SRR334289 | Muscles, sampled at time 10d, exposure to air |
| *Crassostrea gigas* | Transcriptomic | SRR11939888 | Hemocytes, CO_2_ exposure for long time |
| *Crassostrea gigas* | Transcriptomic | SRR11939889 | Hemocytes, CO_2_ exposure for long time |
| *Crassostrea gigas* | Transcriptomic | SRR11939890 | Hemocytes, CO_2_ exposure for long time |
| *Crassostrea gigas* | Transcriptomic | SRR11939891 | Hemocytes, CO_2_ exposure for short time |
| *Crassostrea gigas* | Transcriptomic | SRR11939892 | Hemocytes, CO_2_ exposure for short time |
| *Crassostrea gigas* | Transcriptomic | SRR11939893 | Hemocytes, CO_2_ exposure for short time |
| *Crassostrea gigas* | Transcriptomic | SRR11939894 | Hemocytes, without CO_2_ exposure |
| *Crassostrea gigas* | Transcriptomic | SRR11939895 | Hemocytes, without CO_2_ exposure |
| *Crassostrea gigas* | Transcriptomic | SRR11939896 | Hemocytes, without CO_2_ exposure |
| *Crassostrea hongkongensis* | Transcriptomic | SRR7777763 | Hong Kong oyster, salinity for place 1 |
| *Crassostrea hongkongensis* | Transcriptomic | SRR7777764 | Hong Kong oyster, salinity for place 2 |
| *Crassostrea hongkongensis* | Transcriptomic | SRR7777765 | Hong Kong oyster, salinity for place 3 |
| *Crassostrea hongkongensis* | Transcriptomic | SRR7777766 | Hong Kong oyster, salinity for place 4 |
| *Crassostrea hongkongensis* | Transcriptomic | SRR7777767 | Hong Kong oyster, salinity for place 5 |
| *Crassostrea hongkongensis* | Transcriptomic | SRR7777768 | Hong Kong oyster, salinity for place 6 |
| *Crassostrea virginica* | Transcriptomic | SRR14150612 | Louisiana, acclimated under 10 ℃ |
| *Crassostrea virginica* | Transcriptomic | SRR14150611 | Louisiana, acclimated under 10 ℃ |
| *Crassostrea virginica* | Transcriptomic | SRR14150610 | Louisiana, acclimated under 10 ℃ |
| *Crassostrea virginica* | Transcriptomic | SRR14150609 | Louisiana, acclimated under 10 ℃ |
| *Crassostrea virginica* | Transcriptomic | SRR14150598 | Louisiana, acclimated under 20 ℃ |
| *Crassostrea virginica* | Transcriptomic | SRR14150597 | Louisiana, acclimated under 20 ℃ |
| *Crassostrea virginica* | Transcriptomic | SRR14150618 | Louisiana, acclimated under 20 ℃ |
| *Crassostrea virginica* | Transcriptomic | SRR14150617 | Louisiana, acclimated under 20 ℃ |
| *Crassostrea virginica* | Transcriptomic | SRR14150620 | Louisiana, acclimated under 30 ℃ |
| *Crassostrea virginica* | Transcriptomic | SRR14150619 | Louisiana, acclimated under 30 ℃ |
| *Crassostrea virginica* | Transcriptomic | SRR14150608 | Louisiana, acclimated under 30 ℃ |
| *Crassostrea virginica* | Transcriptomic | SRR14150603 | Louisiana, acclimated under 30 ℃ |
| *Crassostrea virginica* | Transcriptomic | SRR14150607 | New Brunswick, acclimated under 10 ℃ |
| *Crassostrea virginica* | Transcriptomic | SRR14150606 | New Brunswick, acclimated under 10 ℃ |
| *Crassostrea virginica* | Transcriptomic | SRR14150605 | New Brunswick, acclimated under 10 ℃ |
| *Crassostrea virginica* | Transcriptomic | SRR14150604 | New Brunswick, acclimated under 10 ℃ |
| *Crassostrea virginica* | Transcriptomic | SRR14150616 | New Brunswick, acclimated under 20 ℃ |
| *Crassostrea virginica* | Transcriptomic | SRR14150615 | New Brunswick, acclimated under 20 ℃ |
| *Crassostrea virginica* | Transcriptomic | SRR14150614 | New Brunswick, acclimated under 20 ℃ |
| *Crassostrea virginica* | Transcriptomic | SRR14150613 | New Brunswick, acclimated under 20 ℃ |
| *Crassostrea virginica* | Transcriptomic | SRR14150602 | New Brunswick, acclimated under 30 ℃ |
| *Crassostrea virginica* | Transcriptomic | SRR14150601 | New Brunswick, acclimated under 30 ℃ |
| *Crassostrea virginica* | Transcriptomic | SRR14150600 | New Brunswick, acclimated under 30 ℃ |
| *Crassostrea virginica* | Transcriptomic | SRR14150599 | New Brunswick, acclimated under 30 ℃ |
